# Human pericardial macrophages suppress cardiac fibrosis through cystatin C signaling after myocardial infarction

**DOI:** 10.1172/jci.insight.202383

**Published:** 2026-07-16

**Authors:** Ali Fatehi Hassanabad, Sarthak Sinha, Arzina Jaffer, Darrell Belke, Nicole L. Rosin, Elodie Labit, Daniel Young, Friederike I. Schoettler, Keerthana Chockalingam, Benjamin Haeyul Lee, Jameson A. Dundas, Emilie de Chantal, Carmina A. Isidoro, Alexander Tam, Hanjoo B. Shim, Anna N. Zarzycki, Afshin Derakhshani, Elisabeth Gorgiogianni, Jeannine D. Turnbull, Antoine Dufour, Shalina S. Ousman, Jeff A. Biernaskie, Paul W.M. Fedak, Justin F. Deniset

**Affiliations:** 1 Section of Cardiac Surgery, Department of Cardiac Sciences, Libin Cardiovascular Institute, Cumming School of Medicine;; 2 Department of Comparative Biology and Experimental Medicine;; 3 Libin Cardiovascular Institute;; 4 Department of Physiology and Pharmacology, Cumming School of Medicine;; 5 Department of Biochemistry and Molecular Biology, Cumming School of Medicine;; 6 McCaig Institute for Bone and Joint Health, Cumming School of Medicine;; 7 Snyder Institute for Chronic Diseases, Cumming School of Medicine;; 8 Department of Clinical Neurosciences and Department of Cell Biology and Anatomy, Cumming School of Medicine;; 9 Hotchkiss Brain Institute; and; 10 Alberta Children’s Hospital Research Institute; University of Calgary, Calgary, Alberta, Canada.

**Keywords:** Cardiology, Inflammation, Fibrosis, Macrophages

## Abstract

The pericardium plays an important homeostatic role for the neighboring heart, providing both lubrication and structural support. In vivo models have further identified a protective role for the pericardium in modulating cardiac remodeling following myocardial infarction, possibly through the actions of tissue-resident pericardial macrophages. Using patient-derived pericardial samples, we establish that human pericardial immune cells directly inhibit cardiac fibroblast fibrotic activity, and this action is dampened following myocardial infarction. Using single-cell RNA sequencing of patient pericardial fluid cells, we identify two pericardial macrophage subsets that are uniquely altered in response to myocardial infarction, which contributes to a shift in their effector molecule expression profiles. We confirm that fibronectin-expressing human pericardial macrophages are the primary driver of the pericardial antifibrotic actions through the release of cystatin C. Finally, we establish cystatin C as a myeloid cell–derived cardioprotective effector molecule in an in vivo model of myocardial infarction. Collectively, we uncover a molecular mechanism of the local immune environment that regulates cardiac remodeling after myocardial infarction.

## Abstract 2

Special immune cells that surround the heart release a factor that prevents scarring following a heart attack.

## Graphical abstract

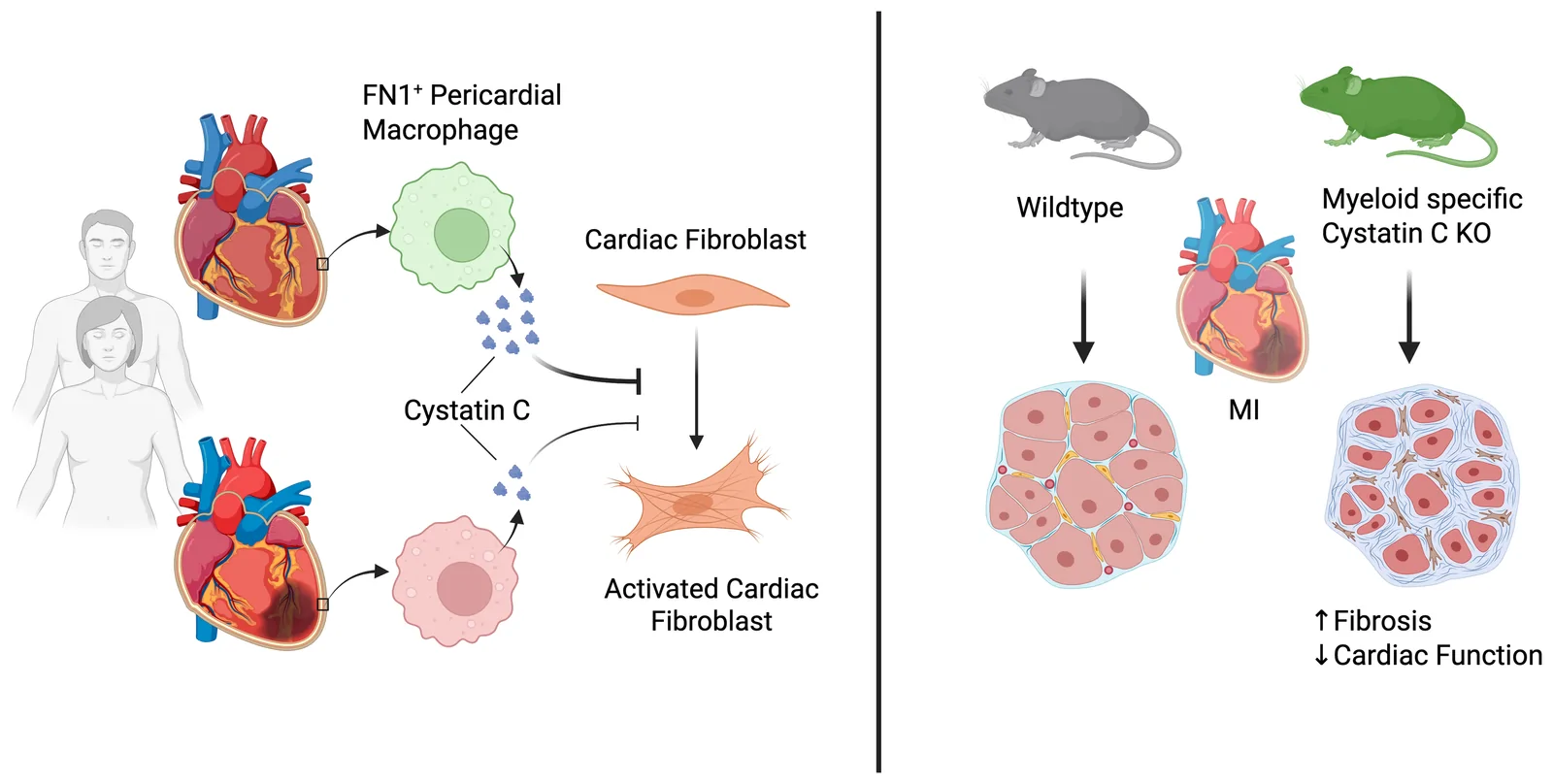

## Introduction

Ischemic heart disease results in progressive and unrelenting changes in cardiac tissues, including the excessive deposition of extracellular matrix products that manifest as a fibrotic scar (1). This remodeling process can lead to the onset of heart failure. The immune response to ischemia-induced myocardial injury is critical to this remodeling process, regulating angiogenic and fibrotic processes (2). Macrophages are vital immune regulators, as they polarize within the local milieu, transitioning from proinflammatory to reparative phenotypes (2). Studies applying single-cell RNA sequencing (scRNA-seq) and spatial sequencing technologies to the human heart have uncovered a diverse repertoire of immune cells, including macrophages, that interact with cardiac-specific cells and influence disease progression (3–5). Understanding these effector mechanisms and how the local environment influences these pathways is essential for developing targeted therapeutic strategies that can be used to modulate cardiac remodeling beneficially and, in turn, blunt the progression of cardiac fibrosis.

The pericardium surrounding the heart is an often overlooked anatomic component of the cardiac environment. Pericardial fluid (PF) within the pericardial space has a vital lubricating function for the dynamic cardiac structures. Using a large-animal model, we have shown that the removal of PF can contribute to pericardial adhesion formation (6), suggesting that PF contains important homeostatic bioactive mediators including growth factors, cytokines, and chemokines (7). These biopeptides can regulate cardiac function via different mechanisms and trigger dysregulation in various disease states (8). Human pericardial immune populations have been identified as sources of these mediators and are responsive to local environmental changes following myocardial injury (9–14). However, the direct contributions of specific immune populations to cardiac homeostasis and remodeling remain largely unclear.

In experimental myocardial infarction (MI) models, we and others have shown that maintaining an intact pericardium is beneficial to preserve cardiac function after myocardial tissue injury (15, 16). This is accompanied by dynamic immune cell changes following cardiac injury and includes the relocation of Gata6-expressing pericardial macrophages to the epicardial surface of the heart (15, 16). These pericardial macrophages have been associated with regulation of fibrosis after MI in some studies (15, 17), while not in others (16). Such a discrepancy highlights the need for further characterization of the pericardial immune environment and identification of mechanisms by which the pericardial immune compartment contributes to the remodeling heart.

This study shows that the human pericardial immune cell compartment has an intrinsic antifibrotic action that is altered following MI. To explore the immune populations responsible, we applied scRNA-seq to cells from patients with a recent MI and compared the expression signature to that in patients without coronary artery disease (CAD). We identified that specific and unique pericardial macrophage subsets are the most perturbed populations following MI. Furthermore, we showed that FN1^+^ pericardial macrophages are responsible for this antifibrotic function via secretion of cystatin C (CST3). Importantly, the MI environment dampened this function through decreased CST3 production. We validated the pathway in vivo, showing that myeloid-specific deletion of CST3 results in an exaggerated fibrotic response after MI and loss of function. Taken together, we show MI-specific alterations of the human pericardial environment and their influence on fibrotic mechanisms in the heart.

## Results

### Human pericardial immune cells inhibit fibroblast activity.

**Figure 1:**
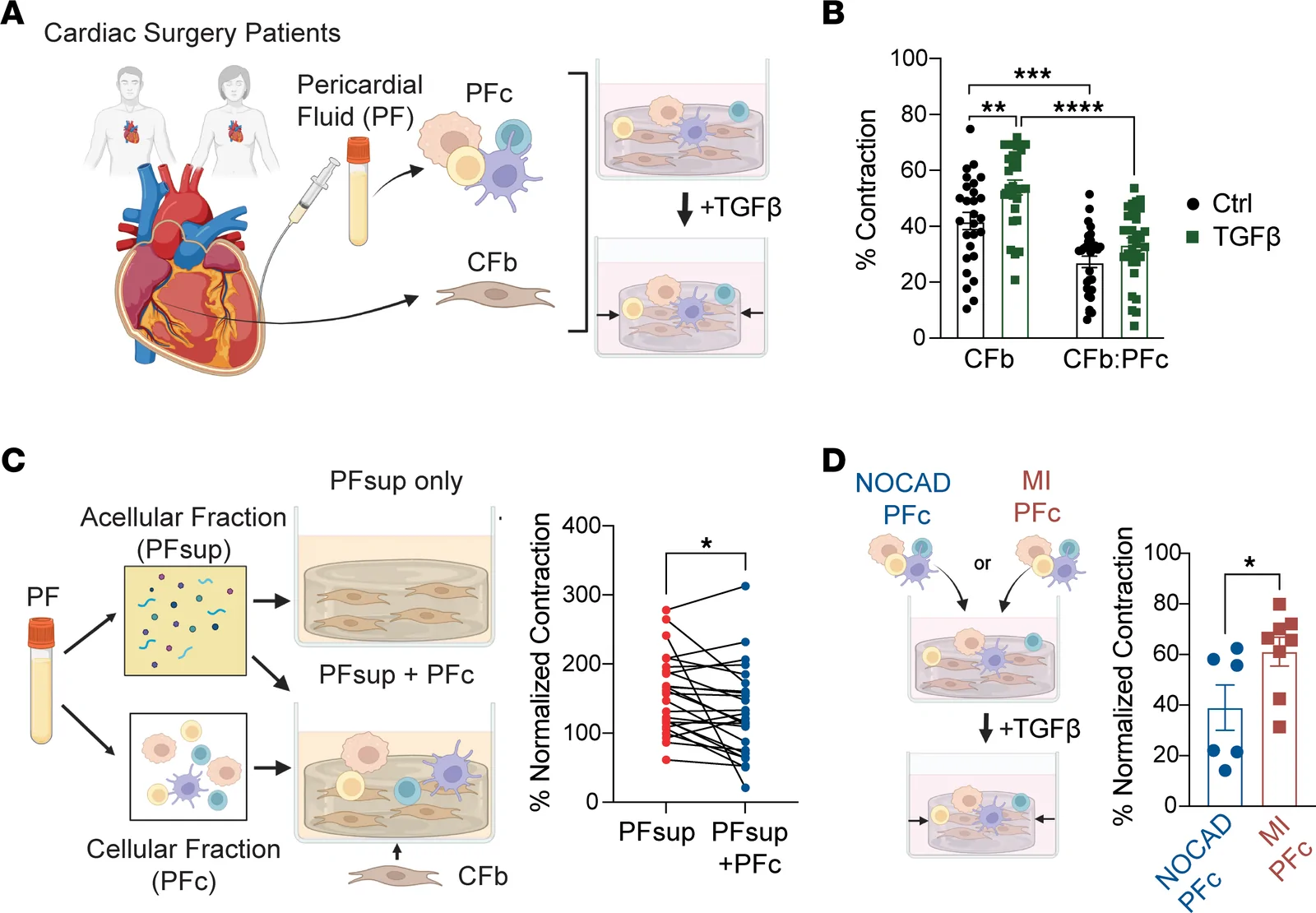
MI elicits a distinct inflammatory response in the pericardial space. (**A** and **B**) Schematic of in vitro coculture setup (**A**) and quantification of cardiac fibroblast–induced (CFb-induced) collagen gel contraction in response to TGF-β_1_ stimulation in the absence (CFb) or presence of human PF cells (PFc) (CFb:PFc) (**B**). *n* = 31 patient pericardial samples and 27 independent experiments. **P < 0.01, ***P < 0.001, ****P < 0.0001, 1-way ANOVA with Tukey’s multiple-comparison test. (**C**) Quantification of CFb-induced collagen gel contraction in response to acellular PF without PFcs (PFsup) or addition of PFcs (PFsup + PFc) from the matching patient. Normalized to CFb contraction in culture medium. *n* = 23 patient pericardial samples and independent experiments. *P < 0.05, paired 2-tailed *t* test. (**D**) Quantification of CFb-induced collagen gel contraction in response to TGF-β_1_ stimulation in the presence of PFcs from NOCAD or MI patients. Normalized to CFb-alone (without PFc) contraction in response to TGF-β_1_ stimulation. *n* = 6 NOCAD patient pericardial samples; *n* = 8 MI patient pericardial samples, and 14 independent experiments. *P < 0.05, unpaired 2-tailed *t* test.

We previously showed that the acellular fraction of PF supernatant (PFsup) can promote cardiac fibroblast activation via TGF-β signaling (10). Since pericardial immune cells can also contribute to the local milieu through secretion of local factors (10), we hypothesized that these immune cells could directly contribute to this fibrotic signaling. To explore this, an established 3D human cardiac fibroblast gel contraction assay was adapted to a coculture system with patient-derived pericardial immune cells (PFcs) (Figure 1A) (18). Human cardiac fibroblasts seeded alone are responsive to TGF-β_1_ stimulation, resulting in increased gel contraction, with the degree of contraction serving as a surrogate for extent of fibroblast fibrotic activity (Figure 1B). Surprisingly, coculturing with patient-derived PFcs resulted in reduced baseline fibroblast activity and blunted TGF-β_1_–induced fibroblast activation (Figure 1B). To further confirm this action of PFcs in their natural context, we added an equivalent number of PFcs back to their respective acellular pericardial fraction (PFsup). PFc numbers in human PF vary between 3 × 10^5^ and 50 × 10^5^ cells/mL; thus a standardized 8 × 10^5^ cells/mL was used to mimic the median value noted in native PF. The re-addition of PFcs blunted the fluid’s fibroblast activation capacity (Figure 1C). Next, we compared the antifibrotic capacity of PFcs from different patient groups and found that PFcs from patients with no flow-limiting coronary artery disease (NOCAD) exhibited a more substantial antifibrotic capacity than patients who had a recent myocardial infarction (MI group) (Figure 1D). These results show that PFcs have an antifibrotic activity that is inhibited in response to myocardial injury.

### MI promotes phenotypic shifts in subsets of human pericardial macrophages.

**Figure 2:**
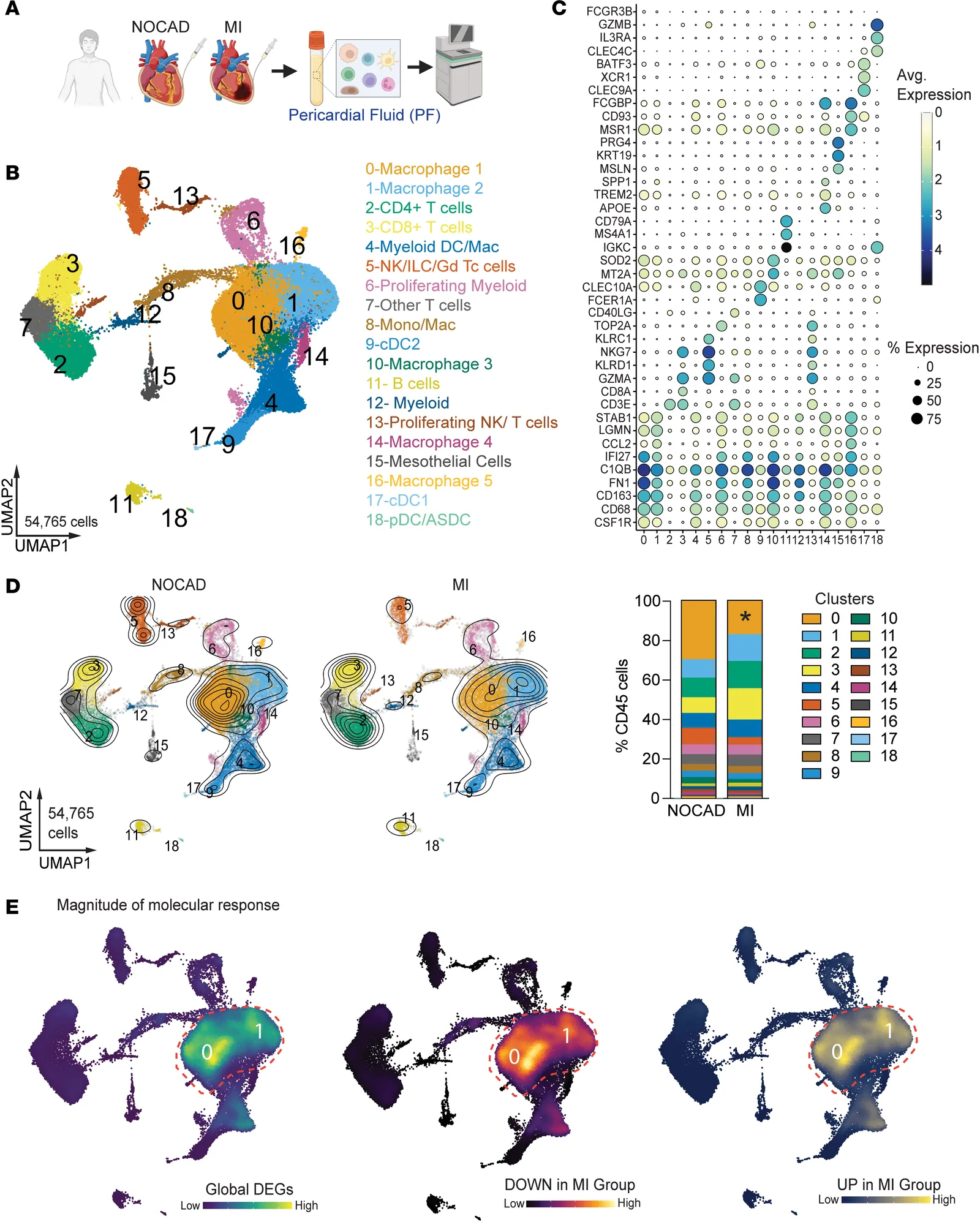
MI promotes phenotypic changes in specific subpopulations of pericardial macrophages. (**A**) Schematic of PF collection from the 2 patient groups, NOCAD (*n* = 4) and MI (*n* = 4), and subsequent analysis using scRNA-seq. (**B**) UMAP projection of 54,765 PFcs from the 8 patients, identifying 19 distinct states/populations of immune and non-immune cells. (**C**) Bubble plot with scRNA-seq expression of canonical lineage and subset markers. (**D**) UMAP projections of PFcs from NOCAD and MI patient samples and stacked plot quantification of cell clusters from NOCAD and MI patient samples. *P < 0.05, generalized linear mixed-effects model. (**E**) Kernel density estimates depicting magnitude of molecular response elicited by pericardial immune cell subsets in both patient groups (left) and those upregulated (right) compared with downregulated (middle) in the MI group relative to the NOCAD group, calculated by summing of differentially expressed gene fold changes for each cell cluster.

To explore the immune cell populations within the PF that contribute to this antifibrotic response and are altered following MI, we generated a queryable atlas of scRNA-seq data for PFcs collected from patients undergoing coronary artery bypass surgery (MI group, *n* = 4) and mitral valve surgery (NOCAD group, *n* = 4) (Figure 2A and Supplemental Table 1; supplemental material available online with this article; https://doi.org/10.1172/jci.insight.202383DS1). Using uniform manifold approximation and projection (UMAP), we analyzed these scRNA-seq data from all 8 patient samples (a total of 54,765 cells). We identified 19 distinct immune and non-immune cell populations/states (Figure 2B). We next confirmed cluster identity by plotting known lineage markers in addition to unique markers (Figure 2C and Supplemental Table 2). We identified 5 distinct macrophage clusters, each exhibiting the canonical macrophage markers *CSF1R*, *CD68*, and *CD163* (Figure 2C). Macrophage subtypes were distinguished by specific expression profiles: cluster 0 and 10 macrophages (*FN1*), cluster 1 and 16 macrophages (*CCL2*), cluster 14 macrophages (*TREM2*, *APOE*, and *SPP1*), and cluster 6 proliferating macrophages (*TOP2A* and *MKI67*). Cluster 10 macrophages were further distinguished from cluster 0 by the expression of *MT2A* and *SOD2*, while cluster 16 macrophages were differentiated from cluster 1 macrophages by the expression of *MSR1*, *CD93*, and *FCGBP*. Among dendritic cells (DCs), cDC2 (cluster 9, *FCER1A*, *CLEC10A*) and myeloid DCs (cluster 4, *CD68*, *CD163*, *CLEC10A*) were the most abundant, with smaller populations of cDC1 (cluster 17, *CLEC9A*, *XCR1*, *BATF3*) and plasmacytoid DCs (pDCs)/AXL^+^SIGLEC6^+^ DCs (ASDCs) (cluster 18, *CLEC4C*, *IL3RA*, *GZMB*). The lymphoid compartment consisted primarily of CD4^+^ T cells (cluster 2, *CD3E*), memory-like CD4^+^ T cells (cluster 7, *CD3E*, *CD40LG*), and CD8^+^ T cells (cluster 3, *NKG7*, *GZMA*). Additional immune populations included innate lymphoid cells and γδ T cells (cluster 5, *KLRD1*, *NKG7*, *KLRC1*), B cells (cluster 11, *IGKC*, *MS4A1*, *CD79A*), and proliferating T/NK cells. Mesothelial cells (cluster 15, *MSLN*, *KRT19*, *PRG4*) were the only non-immune cell type in the PF. The absence of *FCGR3B* expression across all clusters confirms the low abundance of neutrophils.

Comparison of these populations under different disease states revealed that macrophage cluster 0 was proportionally reduced in MI patient samples compared with NOCAD patient samples (Figure 2D and Supplemental Figure 1). Further, the global magnitude of molecular responses between the NOCAD and MI cohorts was largely concentrated within macrophage clusters 0 and 1 with 139 and 133 differentially expressed genes, respectively (Figure 2E and Supplemental Table 3). This is in contrast to the other macrophage clusters 10, 14, and 16, which displayed 9, 3, and 0 differentially expressed genes, respectively (Supplemental Table 3). Pathway analysis for differentially expressed genes in cluster 0 macrophages identified pathways related to interleukin signaling, hemostasis, and angiogenesis, enriched in the MI condition. In contrast, protein/peptide processing and phagocytosis were favored in the NOCAD setting (Supplemental Figure 2). Concerning cluster 1 macrophages, pathways related to allograft rejection and to complement were highlighted under NOCAD conditions (Supplemental Figure 2). In response to MI, cell stress responses and leukocyte differentiation–associated pathways were increased. In the MI setting, both macrophage clusters showed alterations in pathways related to antigen processing and to MHC class II antigen presentation (Supplemental Figure 2).

### Human pericardial macrophage trajectories are responsive to cardiac injury.

**Figure 3:**
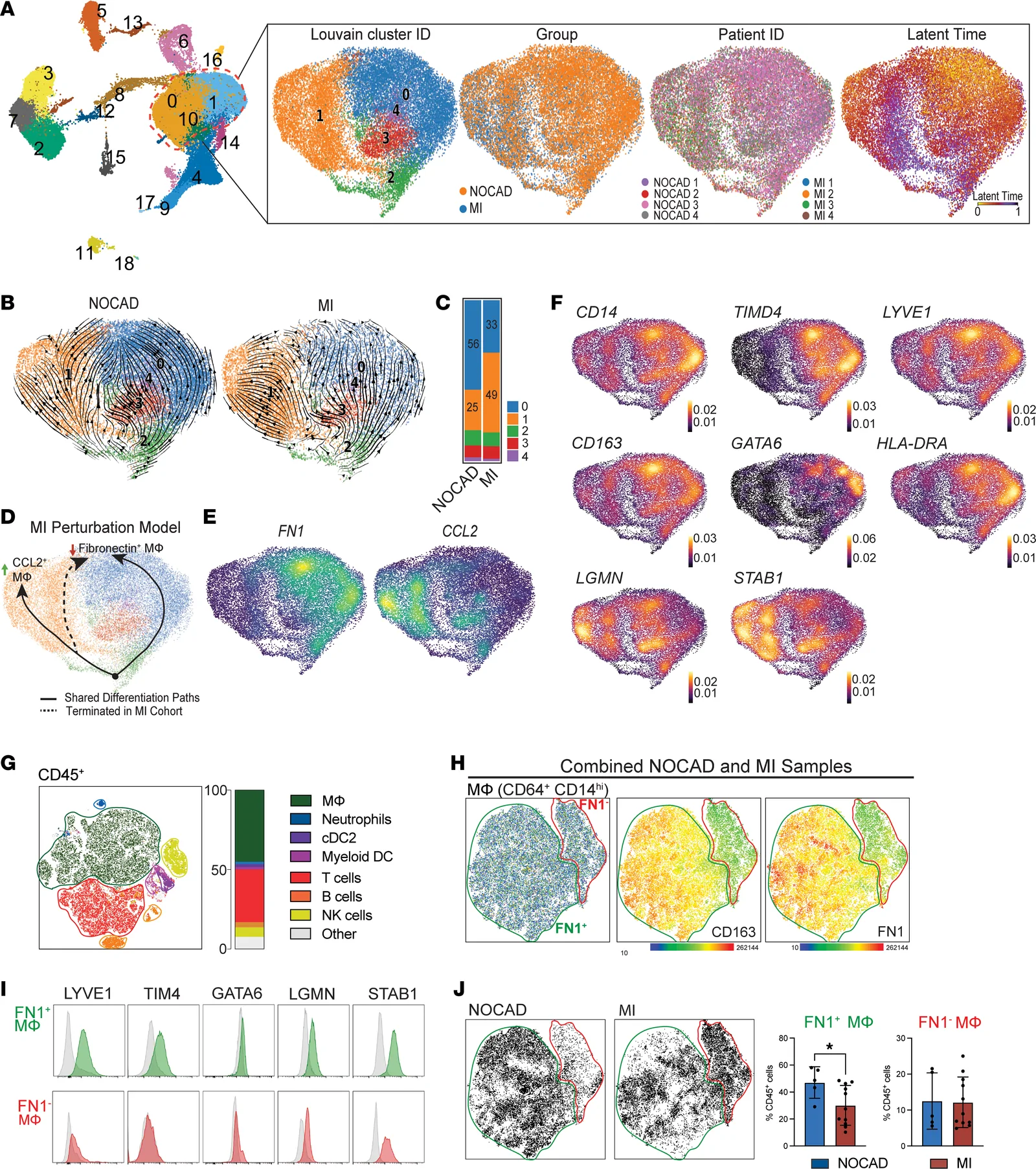
MI dictates maturation trajectories for pericardial macrophages. (**A**) Louvain clustering of pericardial macrophages represented by state, disease group, and individual patient and overlaid with velocity vector fields to generate a latent time plot. (**B**) UMAP plotting RNA velocity analysis of subclustered pericardial macrophages undergoing state transitions, colored by cluster ID. (**C**) Stacked bar plot depicting cluster composition of clinical cohorts examined. (**D**) UMAP colored by pericardial macrophage clusters and overlaid with summary path curves based on vector fields and pericardial macrophage state composition to determine macrophage states. (**E** and **F**) RNA gene expression of selected genes driving divergent maturation trajectories (**E**) and associated defining markers (**F**). (**G**) Flow cytometry analysis and quantification of pericardial immune populations in combined NOCAD and MI samples. (**H** and **I**) Flow cytometry–based representation of pericardial macrophage populations depicted by t-SNE plots (**H**) and expression of protein markers by histogram plots in FN1^hi^ and FN1^lo^ pericardial macrophage populations (**I**). (**J**) Quantification of FN1^hi^ and FN1^lo^ pericardial macrophage populations in both NOCAD and MI patient samples. *n* = 5 for NOCAD, *n* = 11 for MI. *P < 0.05, unpaired 2-tailed *t* test.

Since cluster 0 and 1 macrophages were the prominent immune cell type that responded to MI, we aimed to define their maturation dynamics by performing velocity analysis. Louvain clustering, disease group, individual patient, and velocity length were overlaid with velocity vector fields to generate a latent time plot (Figure 3A). Analyzing the velocity vectors in the 2 patient cohorts revealed 5 macrophage states (Figure 3B). In the NOCAD patients, macrophage state 0 predominated (Figure 3C). In contrast, MI patients demonstrated a shift to macrophage state 1 (Figure 3C). Modeling the differentiation paths revealed 2 trajectories defined by *FN1* and *CCL2* expression (Figure 3, D and E). The vector lengths in the NOCAD condition were longer and more directional toward the FN1^hi^ state in comparison with MI, supporting more significant differentiation of this macrophage state (Figure 3B). In addition, the connection lineage between the CCL2^hi^ state and the FN1^hi^ state was lost in the MI setting (Figure 3D). To characterize these macrophage states/populations further, the gene expression trends of additional markers were queried using kernel density plots. FN1^hi^ macrophages demonstrated higher gene expression of *CD14*, *CD163*, *HLA-DRA*, *TIMD4*, and *LYVE1* regardless of disease context (Figure 3F and Supplemental Figure 3). The expression of the transcription factor *GATA6*, which is commonly used to define resident pericardial macrophages in mice, was also enhanced in the FN1^hi^ macrophages (Figure 3F and Supplemental Figure 3). It should be noted, however, that the overall *GATA6* expression was lower compared with these other markers and *GATA6* was only expressed by approximately 5% of FN1^hi^ macrophages. The CCL2 macrophage population demonstrated higher gene expression of *LGMN* and *STAB1* (Figure 3F and Supplemental Figure 3).

Pericardial macrophage populations were further explored by flow cytometry. Macrophages represented roughly 50% of the local pericardial CD45 compartment, and 2 subsets were identified by the presence and absence of combined CD163 and FN1 expression (Figure 3, G and H). Consistent with transcriptional data, CD163^hi^FN1^+^ macrophages were also defined by TIM4 and LYVE1 expression (Figure 3I). In contrast, CD163^lo^FN1^–^ macrophages did not display CCL2 protein expression (data not shown) nor unique expression of LGMN and STAB1 (Figure 3I). CD163^hi^FN1^+^ pericardial macrophages were enriched in NOCAD patient samples relative to MI patients, whereas CD163^lo^FN1^–^ macrophages remained unchanged, mimicking changes in clusters 0 and 1 observed by scRNA-seq (Figure 2D and Figure 3J).

### Human pericardial macrophage subsets have unique gene regulatory and niche networks.

**Figure 4:**
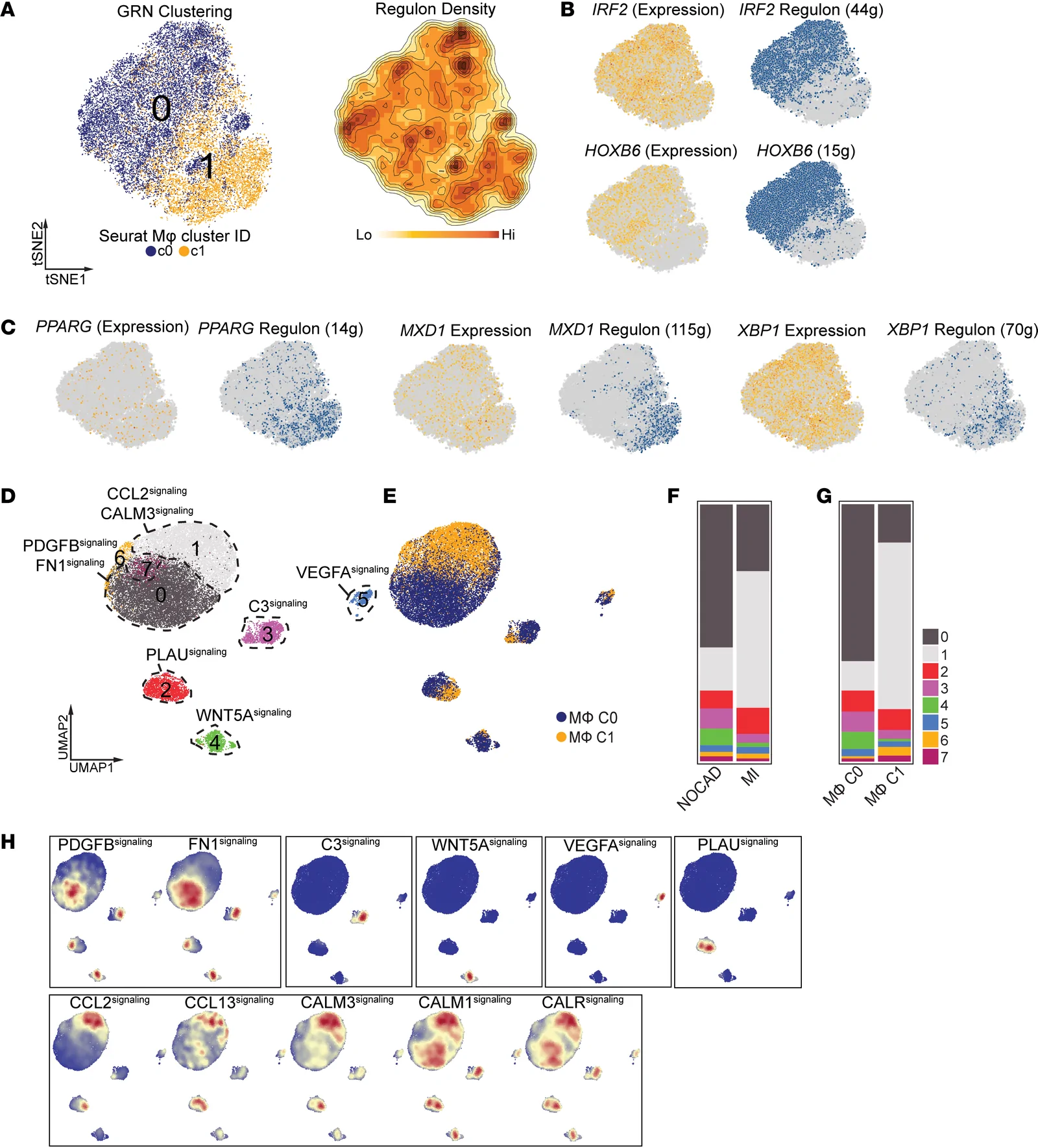
Gene regulatory and signaling networks for pericardial macrophages. (**A**) Gene regulatory network (GRN) clustering and regulon density in cluster 0 (c0) and cluster 1 (c1) pericardial macrophages. (**B** and **C**) Gene regulatory networks preferentially driving cluster 0 (IRF2, HOXB6; **B**) and cluster 1 (PPARG, MXD1, XBP1; **C**) pericardial macrophage profiles. (**D** and **E**) UMAP plotting of signaling pathways associated with pericardial macrophages by NICHES analysis (**D**) and representation of cluster 0 and 1 pericardial macrophages within these signaling clusters (**E**). (**F** and **G**) Stacked bar plots depicting signaling cluster representation between disease states (**F**) or pericardial macrophage clusters (**G**). (**H**) UMAP plotting of expression distribution for PDGFB and FN1; CCL2, CCL13, CALM3, CALM1, and CALR; C3; WNT5A; VEGFA; and PLAU.

To determine the factors potentially driving these macrophage trajectories, gene regulatory network development using single-cell regulatory network inference and clustering (SCENIC) was performed (Figure 4A) (19). The regulon density was also assessed for the 2 macrophage clusters (Figure 4A). This analysis revealed that the transcription factors *IRF2* and *HOXB6* are predicted as key drivers of FN1^hi^ cluster 0 macrophage–related genes (Figure 4B and Supplemental Figure 4). Meanwhile, predicted genes downstream of *PPARG*, *MXD1*, and *XBP1* activity primarily localized to CCL2^hi^ cluster 1 macrophages (Figure 4C and Supplemental Figure 4). These findings suggest that the divergently activated macrophage states are controlled by distinct regulatory programs, uncovering key upstream regulators that dictate pericardial macrophage populations under homeostatic and pathological conditions.

Given the divergent trajectories and regulatory networks of cluster 0 and 1 pericardial macrophages, we next explored their cell-to-cell signaling capacity locally. Performing niche interactions and communication heterogeneity in extracellular signaling (NICHES) analysis on these macrophage subsets revealed their involvement in several signaling pathways (Figure 4, D and E). *FN1*-, *PDGFB*-, *C3*-, and *WNT5A*-related signaling pathways were enriched under NOCAD conditions and in cluster 0 macrophages (Figure 4, D and F–H). In response to MI, *CCL2*- and *CALM*-related signaling was enhanced largely by cluster 1 macrophages (Figure 4, D and F–H). Both *PLAU* signaling and *VEGFA* signaling were relatively equally represented across disease states and macrophage clusters (Figure 4, D and F–H). This supports largely differing functions for these pericardial macrophage subsets within their local milieu.

### FN1^+^ pericardial macrophages inhibit fibroblast activity via cystatin C secretion.

**Figure 5:**
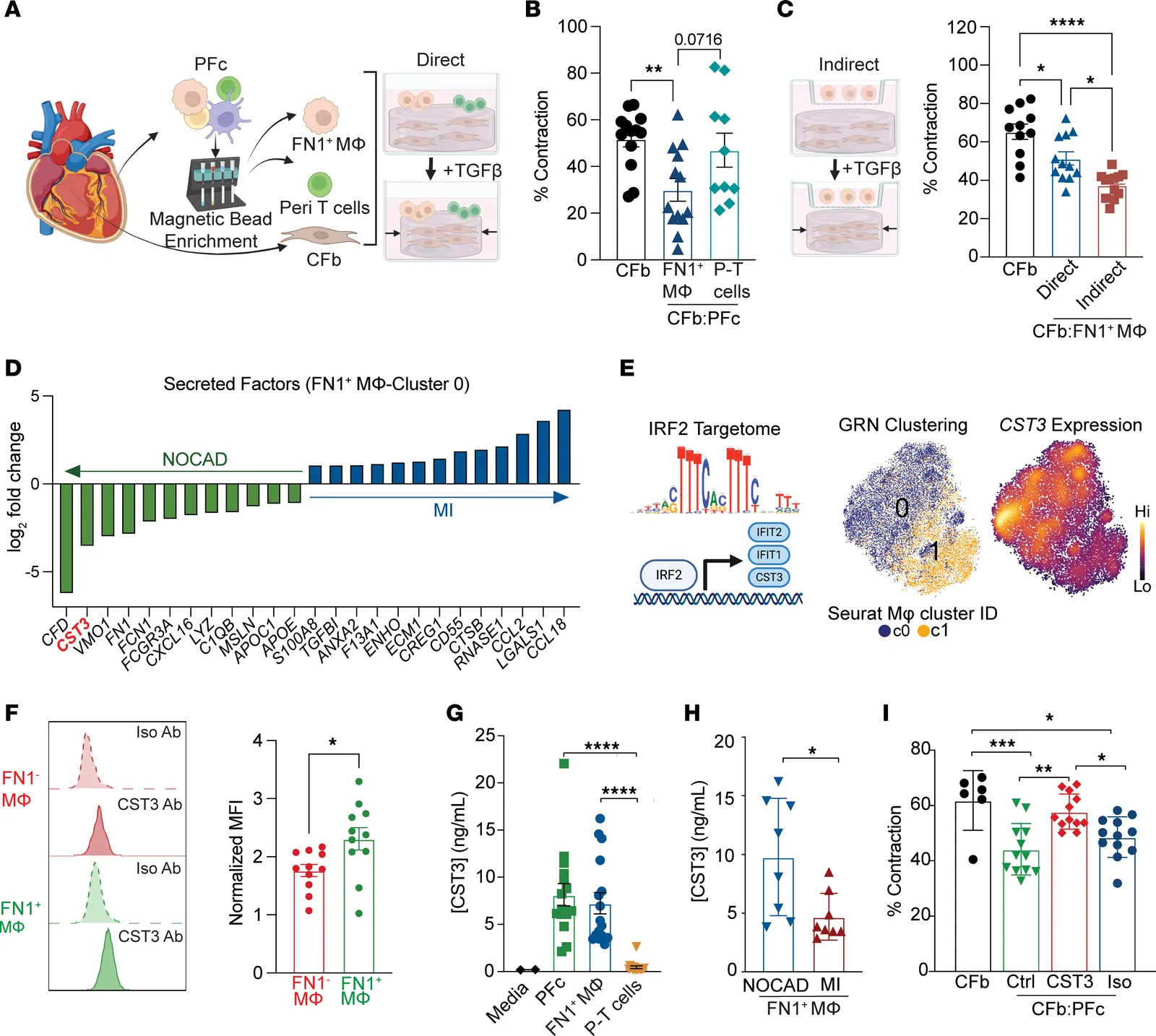
FN1^+^ pericardial macrophages inhibit cardiac fibroblast activity via cystatin C. (**A** and **B**) Schematic of in vitro coculture setup (**A**) and quantification of cardiac fibroblast–induced (CFb-induced) collagen gel contraction in response to TGF-β_1_ stimulation in the absence (CFb) or presence of enriched FN1^+^ pericardial macrophages or enriched pericardial T cells (**B**). *n* = 10 T cell–enriched patient pericardial samples; *n* = 13 FN1^+^ macrophage–enriched patient pericardial samples, and 13 independent experiments. **P < 0.01, 1-way ANOVA with Tukey’s multiple-comparison test. (**C**) Quantification of CFb-induced collagen gel contraction in response to TGF-β_1_ stimulation in the absence (CFb) or presence of direct or indirect (Transwell) pericardial macrophages (CD14^+^). *n* = 12 patient PF samples and 11 independent experiments. *P < 0.05; ****P < 0.0001, unpaired 2-tailed *t* test. (**D**) scRNA-seq quantification of top differentially expressed secreted factors in cluster 0 (FN1^+^) pericardial macrophages in either NOCAD or MI patients. (**E**) Schematic representation of IRF2 target sequence and RNA gene expression of its downstream activated gene, CST3, overlaid on the gene regulatory network clustering for pericardial macrophages. (**F**) Representative histogram plots of CST3 protein expression and quantification of isotype-normalized median fluorescence intensity in FN1^+^CD163^hi^ and FN1^–^CD163^lo^ human pericardial macrophages. *P < 0.05. (**G**) Quantification of CST3 release from conditioned medium of total human PFcs, human pericardial macrophages (CD14^+^), and pericardial T cells (CD3^+^). *n* = 16. ****P < 0.0001, 1-way ANOVA with Tukey’s multiple-comparison test. (**H**) Quantification of CST3 release from conditioned medium of human pericardial macrophages (CD14^+^) from NOCAD and MI patients. *P < 0.05. (**I**) Quantification of CFb-induced collagen gel contraction in response to TGF-β_1_ stimulation in the absence (CFb) or presence of PFc (CFb:PFc) with CST3 or isotype antibody blockade. *n* = 11 patient pericardial samples and 6 independent experiments. *P < 0.05, **P < 0.01, ***P < 0.001, 1-way ANOVA with Tukey’s multiple-comparison test.

Since the FN1^+^CD163^hi^ pericardial macrophage subset is the most abundant cell population and the most altered following MI, we explored whether these cells are responsible for the antifibrotic effects on human cardiac fibroblasts. We applied a magnetic bead approach to isolate FN1^+^CD163^hi^ pericardial macrophages, which resulted in a 90% overall enrichment and 95% among myeloid cells (Supplemental Figure 5). A parallel approach was applied to isolate pericardial T cells, the second most abundant pericardial immune cell type to serve as a cellular control (Supplemental Figure 5). Applying enriched FN1^+^ pericardial macrophages to the coculture system recapitulated the inhibitory effect observed with total PFcs (Figure 1A and Figure 5, A and B). Importantly, this activity was not seen when isolated pericardial T cells were coincubated with the fibroblast gels (Figure 5B). In addition, this macrophage inhibitory action was even enhanced when FN1^+^ pericardial macrophages were seeded on a Transwell insert placed above the fibroblast gel, thus supporting a paracrine mechanism (Figure 5C).

To identify potential soluble mediators that may be responsible for the antifibrotic effects, we explored secreted factors that were differentially expressed between disease settings in cluster 0 (FN1^+^) macrophages. Using a 2-fold change cutoff, we identified 12 factors that were enriched in the NOCAD samples and 13 mediators that were enhanced in the MI samples (Figure 5D). Cystatin C (*CST3*), the second highest differentially expressed mediator in the NOCAD samples, was an intriguing candidate to pursue as the antifibrotic mediator. It is traditionally known as a cysteine protease inhibitor intracellularly but has been reported to inhibit TGF-β signaling through the engagement of TGF-β receptor 1 extracellularly (20). *CST3* was more highly expressed transcriptionally by macrophage cluster 0 (FN1^+^) and is regulated by *IRF2*, identified by the SCENIC analysis as a key driver of this macrophage phenotype (Figure 4B and Figure 5E). Higher protein expression of CST3 in FN1^+^ pericardial macrophages relative to FN1^–^ pericardial macrophages was also confirmed by flow cytometry (Figure 5F). To further validate its active release, we analyzed conditioned media from cultured total PFcs, enriched FN1^+^CD163^hi^ pericardial macrophages, or enriched pericardial T cells. Total PFcs and FN1^+^ pericardial macrophages showed higher release of CST3 than the pericardial T cells (Figure 5G). We also found that FN1^+^ pericardial macrophages from NOCAD patients secreted a significantly higher amount of CST3 compared with MI patient samples (Figure 5H), which is in line with the greater antifibrotic action observed from NOCAD PFcs (Figure 1C). Finally, CST3 antibody blockade in the coculture system abrogated the antifibrotic capacity of PFcs, confirming CST3 as a key mediator of this inhibitory action of pericardial macrophages (Figure 5I).

To determine whether FN1^+^ pericardial macrophages could play a more direct role in the injured heart, we explored whether the observed decrease in FN1^+^ macrophages in PF (Figure 2D) is complemented by the presence of FN1^+^ macrophages in the human heart acutely following MI. For this, we utilized the single-nucleus RNA-seq (snRNA-seq) dataset from Kuppe et al. (21), who sampled left ventricular (LV) cardiac tissue from healthy (control) and ischemic patients at both early (ischemic, <12 days after MI) and late time points (fibrotic, >30 days after MI). FN1^+^ macrophages were enriched as part of the SPP1^+^ macrophage cluster and were primarily found in the ischemic regions of the heart relative to the healthy myogenic or fibrotic tissue (Supplemental Figure 6, A–C). Consistent with the downregulation in *CST3* observed in the FN1^+^ pericardial macrophages, FN1^+^ macrophages in the heart displayed lower expression of *CST3* when compared with resident cardiac macrophage and monocyte/DC clusters.

### Myeloid-derived Cst3 alters fibrotic activity in vivo.

**Figure 6:**
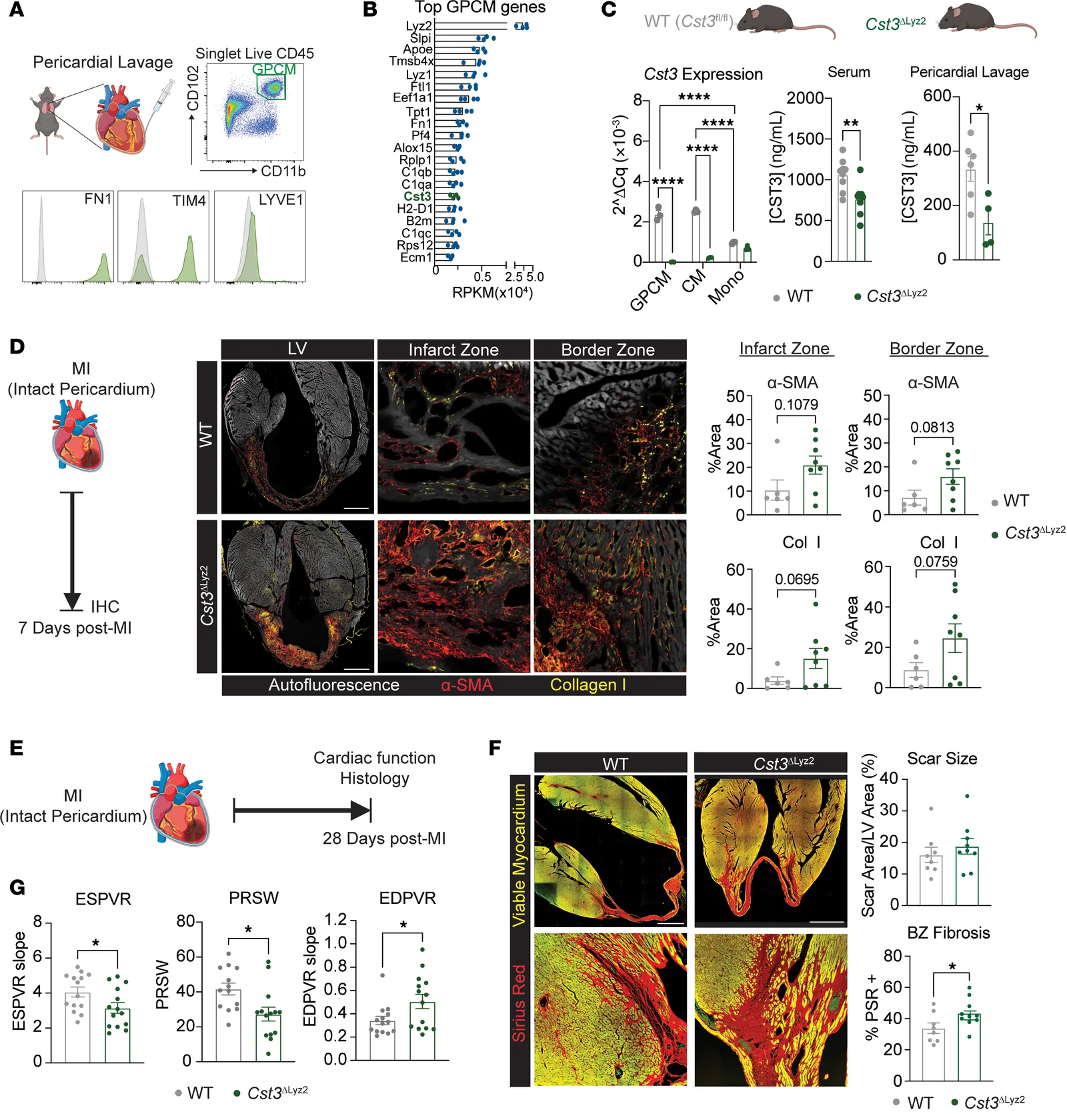
Myeloid-derived *Cst3* alters cardiac fibrosis. (**A**) Representative flow cytometry analysis of mouse GPCM expression of FN1, TIM4, and LYVE1. Representative of *n* = 4. (**B**) RNA-seq quantification of top-expressed genes by sorted mouse GPCMs. *n* = 4. (**C**) qPCR quantification of *Cst3* mRNA expression in pericardial macrophages, cardiac macrophages, and Ly6C^hi^ monocytes from WT (*Cst3^fl/fl^*) and *Cst3*^ΔLyz2^ mice (left), and ELISA quantification of serum (middle) and pericardial lavage (right) CST3 levels from WT and *Cst3*^ΔLyz2^ mice. *n* = 3 for qPCR; *n* = 8 for serum samples; *n* = 6 and 4 for pericardial lavage samples from WT and *Cst3*^ΔLyz2^ mice, respectively. *P < 0.05, **P < 0.01, unpaired 2-tailed *t* test for ELISA; ****P < 0.0001, 1-way ANOVA with Tukey’s multiple-comparison test for qPCR. (**D**) Immunohistochemistry staining and quantification for α-smooth muscle actin (α-SMA) and collagen I (Col I) in the infarct and border zones from WT (*Cst3^fl/fl^*) and *Cst3*^ΔLyz2^ mice at 7 days after MI. Scale bars: 1,000 μm. Data are represented as mean ± SEM; *n* = 6 and 8 for WT and *Cst3*^ΔLyz2^ mice, respectively. Unpaired 2-tailed *t* test, non-parametric (Mann-Whitney) for α-SMA and parametric (Welch’s correction) for Col I. (**E**) Schematic of experimental timeline for cardiac function and fibrosis analysis at 28 days after MI. (**F**) Representative confocal composite stitch images of the LV and border zone (BZ) with Picrosirius red (PSR) staining and quantification of total LV scar size and BZ fibrosis indicated by PSR staining in cardiac cross sections at 28 days after MI for WT and *Cst3*^ΔLyz2^ mice. Scale bars: 1,000 μm. Data are represented as mean ± SEM; *n* = 8 and 10 for WT and *Cst3*^ΔLyz2^ mice, respectively. *P < 0.05, 2-tailed *t* test. (**G**) LV functional parameters (end systolic pressure volume relationship [ESPVR], preload recruitable stroke work [PRSW], and end diastolic pressure volume relationship [EDPVR]) at 28 days after MI for WT and *Cst3*^ΔLyz2^ mice measured by pressure-volume assessment. Data are represented as mean ± SEM; *n* = 14 for WT and *n* = 15 for *Cst3*^ΔLyz2^ mice. *P < 0.05, unpaired 2-tailed *t* test.

The observed antifibrotic property of pericardial macrophage–derived CST3 in vitro prompted the exploration of CST3 function in vivo following MI. We first determined whether pericardial macrophages in the mouse system shared features with those described in patient samples. The mouse system has 2 pericardial macrophage populations; the major population is defined by its expression of the transcription factor Gata6. Gata6-expressing pericardial macrophages (GPCMs) denote high protein expression of FN1 and TIM4, and to a lesser extent LYVE1 (Figure 6A). Further, *Fn1* and *Cst3* were among the top-expressed genes from an RNA-seq dataset of sorted GPCMs (Figure 6B) (15).

To investigate a role of CST3 in vivo, a mouse with conditional deletion of *Cst3* under the control of the lysozyme 2 promoter–driven Cre recombinase (*Cst3*^ΔLyz2^) was generated. This construct contributed to a decrease in *Cst3* expression in pericardial macrophages in addition to cardiac macrophages in *Cst3*^ΔLyz2^ mice compared with *Cst3^fl/fl^* (WT) littermate controls (Figure 6C). Ly6C^hi^ monocytes demonstrated a reduced basal expression of *Cst3* relative to these macrophage populations and remained unchanged in the conditional knockout system (Figure 6C). CST3 protein levels in circulation and pericardial lavage fluid were also decreased in *Cst3^ΔLyz2^* mice relative to WT mice (Figure 6C). Baseline cardiac function was not altered between genotypes (Supplemental Table 4). The loss of *Cst3* in myeloid cells did not impact infarct size after MI induction (48 hours after MI) nor myeloid cell recruitment (day 5 after MI) in comparison with WT littermate mice (Supplemental Figure 7, A and B). To assess whether myeloid-derived *Cst3* could influence myofibroblast generation in vivo, α-smooth muscle actin (α-SMA) and collagen I staining was performed on infarcted cardiac tissue sections collected at day 7 after MI (Figure 6D). Both markers displayed a numerical increase in the infarcted and border zone regions of *Cst3^ΔLyz2^* mice compared with WT controls, although these differences did not reach statistical significance (Figure 6D). To determine the downstream impact of these myofibroblast-linked features, fibrosis and cardiac function were further assessed at 28 days after MI (Figure 6E). The overall scar size did not change; however, increased border zone fibrosis was noted in *Cst3^ΔLyz2^* mice compared with WT littermates (Figure 6F). This was combined with worse cardiac function in *Cst3^ΔLyz2^* mice, including an increase in LV stiffness as indicated by augmented end diastolic pressure volume relationship values and a decrease in the systolic parameters end systolic pressure volume relationship and preload recruitable stroke work (Figure 6G and Supplemental Table 5). Collectively, this establishes a new role for myeloid cell–derived CST3 in modulating cardiac fibrosis in vivo.

## Discussion

Heart failure continues to affect millions of people worldwide (1). Fibrosis remains a significant contributor to cardiac dysfunction and adverse clinical events. The cellular and molecular mechanisms that drive cardiac fibrosis are complex and multifaceted (3). Recent work recognizes the importance of the local immune microenvironment in modulating the initiation, extent, and termination of fibrotic activities (22, 23). Moreover, MI and ischemia secondary to CAD are triggers of cardiac fibrosis (24). Established research identifies circulating cardiac and immune markers in patients affected by cardiac fibrosis and its sequelae (25, 26). However, local biomarkers can also influence fibrotic processes, and require more consideration.

This study builds on our previous research and explores the immune cells within native human PF, showing that acute ischemic events alter the immune cell composition of the pericardial anatomic compartment (14). In the present study, we used single-cell RNA sequencing (scRNA-seq) to provide a more granular characterization of human PF’s cellular compartment. We subsequently compared the scRNA-seq profile between patients with a recent MI and those without CAD. In doing so, we found significant differences in the macrophage population between the two patient groups. These observations can have clinical relevance, as macrophages are accepted to play an important, albeit varied, role in both proinflammatory and pro-reparative processes in the heart (27). Determining the local and systemic factors that drive one phenotype over the other will be important in follow-up studies.

Resident cardiac macrophages are heterogeneous and have a functional impact on cardiac physiology (28). Numerous studies have established an association between macrophages and fibroblasts, underscoring reciprocal interactions that could drive fibrotic processes (29). Macrophage-fibroblast signaling networks are predicted based on the scRNA-seq datasets (7), and emerging work suggests that this network is a therapeutic target in the setting of MI to attenuate cardiac fibrosis (30). However, we have yet to elucidate the functional role of this network. In addition, owing to difficulty obtaining human heart tissues, these previous assessments were done primarily in healthy or end-stage heart failure patients. To better understand the pathways and mechanisms that drive the progression to advanced heart failure, precise evaluation of the macrophage-fibroblast crosstalk at an earlier time interval after myocardial injury is critical.

Our group (15) and others (13) have shown that resident GPCMs can prevent cardiac fibrosis and protect against pericardial inflammation. The present study indicates that human PF contains up to 5 transcriptionally distinct pericardial macrophage populations, with the FN1^+^ macrophages representing the most abundant population and the population displaying an antifibrotic function. Interestingly, this effector function is blunted in response to ischemic injury in the neighboring heart. In addition, our observation that PFcs oppose the natural fibrotic activity of the acellular compartment of PF indicates that these resident FN1^+^ pericardial macrophages may serve as an important homeostatic regulator for the pericardial space. The MI-induced shift in FN1^+^ pericardial macrophages may result in promotion of a local milieu that promotes extracellular matrix remodeling and fibrosis. Local pericardial mediators are likely key drivers for these phenotypic changes. PF-derived small extracellular vesicles from CAD patients were also shown to regulate human pericardial macrophage marker expression, including CD163 (11).

Further, inflammatory mediators such as IL-6 and IL-10, which are also locally enriched in the human PF, are known to induce the expression of CD163 (10, 31). GPCMs in the mouse system have also been shown to relocate from the pericardial space to the thickened epicardial border of the heart, which could impact the nature of the cell interactions and molecule features of the macrophages (15, 16). Reanalysis of the Kuppe et al. snRNA-seq dataset regarding healthy and acute ischemic human cardiac tissue revealed that FN1^+^ macrophages are enriched in the ischemic tissue primarily within an SPP1^+^ macrophage population (21). These SPP1^+^ macrophages are transcriptionally linked to interact with fibroblasts/myofibroblasts within this local environment (21). This appearance in the ischemic heart within 12 days of an MI coincides with the disappearance of FN1^+^CD163^hi^ pericardial macrophages noted previously and in the present study (9). These observations support, but do not definitively prove, the possibility that human pericardial macrophages contribute human macrophage populations within the ischemic heart. Further defining the fate of pericardial macrophages and their interplay with other cells present in their local milieu could provide opportunities to influence the phenotypes of these cells and their effector functions.

Integrating the scRNA-seq analysis and functional differences between PFcs from NOCAD and MI patients helped identify CST3 as a critical effector molecule of pericardial macrophages. CST3 is a protein encoded by the *CST3* gene found in almost all nucleated cells. It is a potent inhibitor of lysosomal cysteine proteases and an extracellular inhibitor of cysteine proteases, which play a pleiotropic role in human vascular pathophysiology. It has been shown to regulate cathepsins, which are overexpressed in atherosclerotic and aneurysmal lesions (32). An extracellular function of CST3 in antagonizing TGF-β signaling in the cancer context is documented (20). Furthermore, CST3 is a critical biomarker for heart and kidney disease, where its concentration is an independent risk factor for heart failure in older adults (33). Moreover, plasma concentrations of CST3 in patients with CAD are associated with a higher risk for secondary cardiovascular events (34). However, the mechanism of action of CST3 in the ischemic heart has not been investigated to date. We establish that FN1^+^ pericardial macrophages are a rich source of CST3 and can regulate myofibroblast activation through their paracrine actions. We also establish that a myeloid deficiency of CST3 contributes to increased cardiac fibrosis after MI. Although GPCMs are identified as a key producer of CST3 in the mouse system, the current targeting approach cannot discount the potential contribution of other myeloid populations. For example, resident cardiac macrophages in both the mouse and the human context also express CST3 and have known antifibrotic effects in the MI context (21, 35). This myeloid-targeted deficiency also contributed to a reduction in both pericardial and circulating levels of CST3, which could also impact these observed effector functions. Future development of a pericardial macrophage–specific deletion approach is needed to better evaluate relative contributions of the various myeloid cell components in this system and on circulating levels of CST3. Given the correlational link between CST3 and heart failure, an outstanding question is whether, in more advanced settings of heart failure, CST3 maintains an antifibrotic function. Further, it remains unclear whether CST3 directly exacerbates disease progression in heart failure. More mechanistic evaluation of CST3 function in other cardiac disease models at both acute and advanced stages would help to delineate the context-dependent functions of this mediator.

Growing evidence supports a critical role for local immune microenvironments coordinating adaptive physiological and maladaptive pathological states. These observations have had translational and clinical importance, including the development of targeted therapeutics, such as checkpoint inhibitors, which have positively impacted patient outcomes. Heart failure is a syndrome that can result from a constellation of different etiologies, but cardiac tissue fibrosis is ubiquitous and central to the progression of the disease. Similarly, cardiac fibrosis is the culmination of a complex cascade of processes, many of which have immune-mediated etiologies. Although extensive research has focused on describing the systemic markers of cardiac fibrosis and its sequelae, little attention has been given to local pericardial factors that can impact fibrotic activity.

The present work aims to bridge the knowledge gap by defining the pericardial immune cell profile and its role in cardiac fibrosis after MI. First, we show that the local versus systemic responses to MI are distinct. This observation may have clinical implications as pericardial mediators can potentially have diagnostic, prognostic, or therapeutic properties. Second, we identify specific subsets of human pericardial macrophages that are perturbed following an MI — a phenomenon that is not present in patients without CAD. This finding supports the potential utility of leveraging pericardial factors for clinical purposes, especially when managing patients presenting with MI. Third, we show that human pericardial macrophages can inhibit fibroblast activation and, more broadly, mouse myeloid cells can blunt cardiac fibrosis via CST3. This discovery is exciting as it offers a strong foundation and a compelling rationale for developing therapeutic agents (such as recombinant CST3) that can be delivered locally and target precise pericardial factors to blunt or attenuate cardiac fibrosis after MI. Future studies should explore whether clinical heart failure can be prevented or reversed by the local administration of CST3 in patients presenting with MI.

## Methods

### Sex as a biological variable.

Our study used human samples from male and female patients, except for the scRNA-seq analysis, which was completed on male samples only. This was simply based on the higher male/female ratio of patients who undergo cardiac surgery at our center and the narrow collection window we had for these samples. Given that results from these scRNA-seq data were further validated by flow cytometry and in vitro functional assays using male and female samples, this would support that these results apply to both sexes. Our study used male and female mice for in vivo experiments.

### Patient sample acquisition.

Patients undergoing cardiac surgeries at Foothills Medical Centre (Calgary, Alberta, Canada) were prospectively enrolled in the study after providing written informed consent. The experiments were conducted under the approval of the Conjoint Health Research Ethics Board at the University of Calgary, under the Declaration of Helsinki (Ethics ID: REB16-1906; approved February 12, 2021). Inclusion criteria were age greater than 18 years and patients undergoing surgery through a conventional full median sternotomy. We excluded patients who received insulin or immunosuppressive medications, patients with a history of inflammatory or rheumatic disease, patients who required dialysis, patients with active infective endocarditis, and those who underwent emergent surgery or redo surgery.

### Animals.

C57BL/6J and *Lyz2^cre^* mice were purchased from The Jackson Laboratory and were bred in-house. The *Cst3*-floxed mice were bred in-house with *Lyz2^cre^* mice. *Lyz2^cre^* *Cst3^fl/fl^* mice were subsequently bred with *Cst3^fl/fl^* to generate Cre^+^ and Cre^–^ littermates. All mice were housed in the specific pathogen–free, double-barrier unit at the University of Calgary. Mice were fed autoclaved rodent feed and water ad libitum.

### Human cardiac myofibroblast isolation.

Human cardiac fibroblasts were isolated from right atrial appendages taken from consenting patients undergoing cardiac surgery at Foothills Medical Centre as previously described (36, 37). In brief, samples were minced into 0.5- to 1-mm fragments and suspended in Iscove’s modified Dulbecco’s medium (IMDM; Lonza) supplemented with 10% fetal bovine serum (Gibco by Life Technologies, Burlington, Ontario, Canada) and 50,000 units of penicillin-streptomycin (Life Technologies). Tissue suspensions were plated and cultured at 37°C in 5% CO_2_. Passage 4 was used for experiments. No cells were pooled, and each experiment was done with a distinct set of fibroblasts; thus, the experimental replicates were completed using fibroblasts harvested from atrial appendages of different patients. All cells were serum-starved for 24 hours before experimental use.

### 3D collagen gel contraction model and coculture system.

The 3D collagen gel contraction model has been previously validated as a functional measure of myofibroblast activity, in which percentage gel contraction positively correlates to myofibroblast activity levels (38–41). Briefly, a collagen gel solution was prepared by combination of the following on ice: 10% 10× minimal essential medium (Gibco); rat tail type I collagen (BD Biosciences) to a final concentration of 1.8 mg/mL; 1 N NaOH to a pH of 7–8; and PBS. Human cardiac myofibroblasts, serum-starved for 24 hours, were incorporated to achieve a concentration of 1.0 × 10^5^ cells per well, and the solution was aliquoted into a 24-well plate (Falcon). Notably, a minimum of 8 patients’ cardiac fibroblasts were used for each experiment, and no pooling of cells was performed. The collagen gel was polymerized at 37°C for 1 hour. Once polymerized, a treatment solution of 500 μL of serum-free medium alone (representing baseline myofibroblast activity) or in combination with recombinant human TGF-β_1_ (TGF-β; Gibco; 10 ng/mL; positive control known to increase myofibroblast activity) was added to each of the collagen gel–containing wells. For the coculture approach, PFcs collected from patients were seeded directly with the human cardiac myofibroblast mixture or on a Transwell (0.4 μm pore) culture insert at a concentration of 4.0 × 10^5^ cells per well and placed above the gel. Cardiac fibroblasts were derived from human right atrial appendage tissue (*n* = 27) as described below. Native PF was collected from 31 patients undergoing cardiac surgery as detailed above. To compare the capacity to induce myofibroblast activation (as quantified by gel contraction) between NOCAD and MI, native PF was collected from 6 NOCAD and 8 MI patients, respectively. For CD14^+^ and CD3^+^ enrichment experiments, cardiac fibroblasts were derived from human right atrial appendage tissue (*n* = 10) as described below. Native PF was collected from 13 patients undergoing cardiac surgery as detailed above. Pericardial CD14^+^ and CD3^+^ cells were enriched and collected using EasySep StemCell (StemCell Technologies). For indirect coculture of cells, cardiac fibroblasts were derived from 11 patients, and native PF was collected from 12 patients and enriched for their CD14^+^ cells. Collagen gels were released from the 24-well plate after 24 hours and imaged an additional 24 hours after release. Images were analyzed using ImageJ (version 1.44g, NIH). Collagen remodeling was indicated by percentage contraction, calculated as follows: % contraction = [(surface area of the well – surface area of the collagen gel)/surface area of the well] × 100.

### scRNA-seq library construction.

PF samples from 4 patients with CAD who presented with an acute coronary syndrome (referred to as the MI group) undergoing coronary artery bypass grafting surgeries and 4 patients with no flow-limiting CAD (referred to as the NOCAD group) undergoing mitral valve surgeries were collected for scRNA-seq analysis. All 8 samples were processed with the 10x Genomics Chromium Single Cell 3′ Reagent Kit (v3 Chemistry) according to the user guide (42). Initially, 100,000 single cells were loaded for partitioning into 10x Genomics NextGEM Gel Bead emulsions (GEMs) to generate full-length cDNA. DynaBeads MyOne Silane magnetic beads (Thermo Fisher Scientific) were used to remove biochemical reagents/primers from the reaction mix, and cDNA was amplified using PCR for 12 cycles. Read 1 primer sequences, P5 primers, P7 primers, i7 sample index, and read 2 primer sequences were added to the cDNA during library construction. The quality and quantity of resulting libraries were analyzed using TapeStation D1000 ScreenTape assay (Agilent). Sequencing occurred using Illumina NovaSeq S2 and SP 100-cycle dual-lane flow cells until the recommended 20,000 reads per cell. All FASTQs were aligned to the standard pre-built GRCh38 reference genome using the Cell Ranger 3.1.0 pipeline. Using Cell Ranger aggr for between-sample normalization, 8 samples that were above alignment quality control measures were aggregated into a single dataset. The aggregated dataset contained a total of 54,765 cells sequenced to 21,280 post-normalization reads per cell.

### scRNA-seq analyses and computational workflows.

The resulting gene barcode matrix from the aggregated dataset was imported and processed using the Seurat (v4) R tool (43) for filtration, normalization, scaling, integration, Louvain clustering, dimensionality reduction, differential expression analysis, and visualization. Cells with abnormal transcriptional complexity (fewer than 200 genes, more than 5,500 genes, fewer than 40,000 UMI, or greater than 25% of mitochondrial reads) were considered low quality and therefore removed from the analysis. Integration of MI and non-CAD patient samples was completed using the CCA and MNM techniques employed by Seurat v4. Cell annotations were assigned by mapping of the aggregated dataset to the recently published peripheral blood mononuclear cell single-cell joint RNA/CITE-seq multiomic reference (43) and known cell signatures.

### Statistical approach for comparing pericardial cell proportions.

To compare pericardial cell proportions between MI and NOCAD PF samples, we used a generalized linear mixed-effects model. Here, the condition (MI versus NOCAD) was treated as a fixed effect and individual patients were treated as a random effect to account for interpatient variability. Model fitting was performed using the Laplace approximation via the glmer function in the lme4 R package (version 1.1-27.1) (44). *P* values for the fixed effect were calculated using the car package (version 3.0-11). Box plots depicting the cell cluster proportions were generated using the ggplot2 package (Supplemental Figure 1).

### Perturbation score calculation.

The differentially expressed genes were calculated with Seurat (v4) (43) by Wilcoxon’s rank-sum test, which had an average log fold change (logFC) greater than 0.25 and an adjusted *P* value less than 0.05. The cumulative logFCs were generated for each Louvain cluster to indicate the discrepant genes on a cluster-by-cluster basis (45). These scores, known as perturbation scores, were then visualized using Nebulosa (v1.0.2) density plots (46).

### Constructing macrophage trajectories using RNA velocity.

The Cell Ranger count-generated BAM files were aligned with the RNA velocity command-line tool using the run10x command and the human (GRCh38) annotations to construct cell type trajectories. The resulting loom files (spliced and unspliced counts) were combined and imported into Seurat through the ReadVelocity function in SeuratWrappers version 0.2.0 and subsequently normalized using SCTransform (v0.3.2) (47). The data underwent dimensionality reduction to yield UMAP projects. Both stochastic and dynamic models for RNA velocity were estimated, and the stochastic model was used as the default for subsequent analyses as both models yielded similar results (48). Kernel density plots were created using ggplot2 (v3.1.1) based on the AnnData metadata to show the velocity-inferred latent time distribution.

### Gene regulatory network analysis using SCENIC.

Single-cell regulatory network inference and clustering (SCENIC) (19) was used to infer the gene regulatory networks and transcription factors that were active in the samples. Cluster 0 and cluster 1 macrophages were subsetted from the scVelo-realigned Seurat object and processed using the default metrics in the SCENIC vignette (https://github.com/aertslab/SCENIC) with the hg19 RcisTarget reference. Group differences between MI and NOCAD were tested using linear mixed-effects models, with group as a fixed effect and patient as a random effect to account for intra-sample correlation. This approach avoids inflated significance from treating cells as independent. Results were visualized using box plots with jittered patient-level points and annotated *P* values.

### Intact pericardium coronary artery ligation surgery.

MI was induced by permanent ligation of the left anterior descending (LAD) coronary artery without excision and tearing of the outer pericardial tissue layer (pericardium intact) (49). For the procedure, mice were anesthetized using isoflurane (2% isoflurane with oxygen as carrier gas), intubated, and ventilated using a VentElite Small Animal Ventilator (Harvard Apparatus). The chest wall was shaved and cleaned with ethanol and iodine before a left thoracotomy in the fourth intercostal space. The LAD coronary artery was ligated with a monofilament 8-0 suture (Ethicon, Johnson & Johnson). The chest and skin were closed with a 5-0 Vicryl suture (Ethicon), and air in the thorax was evacuated via a pleural catheter. Mice were injected with 0.1 mg/kg buprenorphine (s.c.) for analgesic control. The same surgeon performed all procedures in a blinded fashion. For myeloid CST3-deficient mice, experiments were completed in both male and female with no anticipated sex differences based on previous data with this model. A starting sample size of 15 for these experiments was calculated based on a 2-tailed test, an effect based on pilot data, a sigma level of 0.05, a power of 0.9, and an attrition rate of 15%. Data were collected for *n* = 14 and *n* = 15 for the *Cst3^fl/fl^* and *Lyz2^cre^ Cst3^fl/fl^* mouse groups, respectively.

### Cardiac function assessment.

Cardiac function was assessed by pressure volume loop assessment at baseline and 28 days after MI. Mice were anesthetized using isoflurane (4% induction, 2% maintenance), intubated, and ventilated with a VentElite Small Animal Ventilator (Harvard Apparatus). The neck was shaved, cleaned, and excised to expose the right carotid artery. The carotid was occluded distally, and a 1F conductance catheter (Millar Instruments) was gently advanced down the carotid artery into the left ventricular chamber. After recording of baseline pressure volume measurements, an abdominal occlusion of the vena cava was performed to obtain a family of loops with varying afterload and preloads. After recording was complete, a parallel conductance value was obtained by jugular vein injection of hypertonic saline, and blood was withdrawn to calibrate the conductance catheter. Animals were euthanized and tissues collected for subsequent analysis. Data were analyzed using the PV loop analysis module in Labchart (ADI Instruments).

### Histological staining.

For fibrosis assessment, hearts collected after functional assessment at 28 days after MI were fixed in 10% formalin and sent to the Libin Pathology Core for paraffin embedding, sectioning (6 μm), and Picrosirius red (PSR) staining. Composite stitch images of the entire heart section for PSR staining were obtained using a Nikon AXR resonant scan confocal microscope with a ×25 objective. Quantification of fibrotic staining was performed using ImageJ software by first subtracting the green autofluorescent channel from the red channel (PSR staining), then creating a binary thresholded image. Total scar area was calculated as total scar area in the LV/total LV area. Border zone (BZ) fibrosis was calculated as fibrosis area in the BZ/total BZ. The BZ area was defined as the area between where the infarct starts to widen, forming a V shape, and the edge of the main scar where the LV wall returns to a normal thickness.

For immunohistochemistry, hearts were harvested at day 7 after MI and fixed overnight in 4% PFA. Samples were sequentially placed overnight in 15% and then 30% sucrose solutions. Samples were then embedded in OCT compound and sectioned into 10 μm sections before storing at 4°C. Before staining, samples were exposed to open air for 30 minutes. Samples were blocked for 1 hour in a PBS solution containing 2.5% donkey serum (MilliporeSigma) and 0.2% Triton X (MilliporeSigma). Collagen type I antibody (Rockland, 600.401.1030.1, polyclonal) was diluted to a 1:50 concentration and incubated overnight, followed by secondary staining with donkey anti-rabbit CF750 (Biotium, 20828) for 1 hour in a PBS solution containing 2.5% donkey serum and 0.2% Triton X (MilliporeSigma). Samples were then stained using an AF555-conjugated α-SMA antibody (Abcam, AB202509, clone EPR5368) for 1 hour in a PBS solution containing 0.5% donkey serum and 0.2% Triton X. Antifade fluorescence mounting medium with DAPI (Invitrogen) was applied to the sections before coverslip mounting for imaging. Images were acquired on a confocal microscope at ×20 water-immersion magnification with a 6 μm range and 1.5 μm steps. Stitched images of transverse heart sections were generated using NIS-Elements software. Stitched images were denoised in NIS-Elements. For image analysis, infarct and border regions were traced in QuPath software. The infarct area was defined as the region of reduced autofluorescence within the left ventricle. Border zone regions were defined as areas of transitioning myocardial thickness between the remote and infarct areas. Thresholded signal was defined by comparison to secondary-only controls after background subtraction with a rolling ball radius of 5 pixels. Percentage area was calculated by division of the thresholded signal area by the total area of the region of interest.

Cardiac tissue viability was assessed 48 hours after MI by TTC staining. Hearts were collected into heparinized saline. The aorta was then cannulated and the heart retrogradely perfused with 2 mL of saline at a rate of 0.66 mL/min with a syringe pump. After perfusion, the heart was gently dried using a Kimwipe (Kimtech) and wrapped in plastic wrap, placed in a microcentrifuge tube, and stored at –20°C for 15 minutes. The heart was then sectioned into 1 mm slices using a Rodent Heart Slicer Matrix (Zivic Instruments), and slices were placed into room temperature for 3 minutes before being transferred to a rocker at 37°C for 20 minutes. Slices were then transferred to room-temperature 10% buffered formalin for 1 hour before being imaged using a stereomicroscope and a Google Pixel 7 Pro Camera and a NexYZ 3-Axis Universal Smartphone Adapter (Celestron). Before imaging, all tissue slices were weighed. Photographs were analyzed using ImageJ to manually contour infarct, left ventricle, right ventricle, and total tissue area. Infarct size was reported as a percentage of total LV.

### Flow cytometry.

Human PF was collected during cardiac surgery using a syringe before opening of the pericardial cavity. Mouse pericardial lavage was performed in anesthetized animals by a single injection of 100 μL of sterile saline into the pericardial cavity via a PE-10 catheter and subsequent retrieval. The lavage was repeated twice and combined for the same animal. The murine heart ventricular tissue was excised, minced, and subsequently digested in 450 U/mL collagenase I (MilliporeSigma), 125 U/mL collagenase XI (MilliporeSigma), 60 U/mL DNase I (Roche), and 60 U/mL hyaluronidase (MilliporeSigma) with PBS for 1 hour at 37°C on an orbital shaker. Homogenates were then passed through a 40 μm cell stainer and spun down at 300 *g* for 5 minutes at 4°C. Mouse blood was collected through cardiac puncture and incubated with 6% dextran for 30 minutes at room temperature to precipitate out red blood cells. The top clear layer was subsequently centrifuged at 300 *g* for 5 minutes at 4°C. Bone marrow was flushed from femur and tibia bones. Flow-through was collected from bones of the same animal and centrifuged at 300 *g* for 5 minutes at 4°C. Mouse spleen was excised and passed through a 70 μm cell stainer, and cells were pelleted by centrifuging at 300 *g* for 5 minutes at 4°C. For mouse samples, residual red blood cells were lysed using ACK (Invitrogen). Once single-cell suspensions were obtained, cells were blocked using anti–mouse CD16/32 antibody (Bio X Cell, 2.4G2 clone) or human FcγR binding inhibitor (eBioscience) and Ghost Dye Red 710 (TONBO Biosciences) viability stain for 20 minutes. PFcs were then stained for 30 minutes with antibodies. For human samples this included CD45 (BioLegend, 304036, clone HI30), CD64 (BioLegend, 395922, clone 10.1), CD14 (BioLegend, 367110, clone 63D3), CD1c (BioLegend, 331542, clone L161), CD163 (BioLegend, 333611, clone GHI/61), CD16 (BioLegend, 302018, clone 3G8), CD19 (BioLegend, 392506, 4G7), CD56 (BioLegend, 318328, clone HCD56), CD3 (Thermo Fisher Scientific, 48-0037-42, clone OKT3), LYVE1 (Thermo Fisher Scientific, PA1-16635, polyclonal), and TIM4 (BioLegend, 354003, clone 9F4). For mouse samples this included CD45 (BioLegend, 103138, clone 30-F11), CD11b (Thermo Fisher Scientific, 25-0112-82, clone M1/70), CD102 [BD Biosciences, 740346, clone 3C4(mIC2/4)], Ly6G (BioLegend, 127612, clone 1A8), Ly6C (BioLegend, 108425, clone HK1.4), CD64 (BD Biosciences, 558455, clone X54-5/7.1), LYVE1 (Thermo Fisher Scientific, 50-0443-82, clone ALY7), and TIM4 (BioLegend, 130008, clone RMT4-54). Intracellular staining was subsequently performed using the FOXP3 intracellular staining kit (Thermo Fisher Scientific). For human samples this included FN1 (BD Biosciences, 563100, clone 10/fibronectin), GATA6 (Cell Signaling, 5851, clone D61E4), LGMN (R&D Systems, AF2199, polyclonal), STAB1 (R&D Systems, AF3825, polyclonal), and CST3 (R&D Systems, AF1238, polyclonal), and for mouse samples, FN1 (Abcam, ab237286, clone EPR19241-46). Anti-rabbit (BioLegend, 406414, polyclonal; Thermo Fisher Scientific, A11035, polyclonal), anti-goat (Thermo Fisher Scientific, A21085, polyclonal), and anti-sheep (Thermo Fisher Scientific, 31627, polyclonal) IgG secondary antibodies were used for GATA6/LYVE1, LGMN/CST3, and STAB1, respectively. Human samples were run using a BD FACSCanto flow cytometer and analyzed using FlowJo software (Tree Star). Cellular counts were calculated with the inclusion of counting beads (Thermo Fisher Scientific).

CD163^hi^FN1^+^ macrophages were identified as CD45^+^, CD64^+^, CD14^+^, CD1c^–^, CD163^hi^, CD16^hi^, FN1^hi^. FN1^–^ macrophages were identified as CD45^+^, CD64^+^, CD14^+^, CD1c^–^, CD163^lo^, CD16^lo^, FN1^lo^. DC2s were identified as CD45^+^, CD64^int^, CD14^–^, CD1c^+^. Myeloid DCs were identified as CD45^+^, CD64^int/+^, CD14^int^, CD1c^int^. T cells were identified as CD45^+^, CD3^+^. Neutrophils were identified as CD45^+^, CD64^–^, CD16^+^, SSC^hi^. B cells were identified as CD45^+^, CD3^–^, CD64^–^, SSC^lo^, CD16^–^, CD19^+^. NK cells were identified as CD45^+^, CD3^–^, CD64^–^, CD19^–^, CD56^+^, CD16^+/int^. Populations that fell outside these listed gates were collectively noted as “other” cells. For mouse experiments, pericardial macrophages were identified as viable CD45^+^CD11b^+^CD102^+^, cardiac neutrophils were identified as CD45^+^CD11b^+^Ly6G^+^, cardiac monocytes were identified as CD45^+^CD11b^+^Ly6G^–^Ly6C^hi^CD64^lo^, and cardiac macrophages were identified as CD45^+^CD11b^+^Ly6G^–^Ly6C^lo^CD64^+^.

### RNA isolation and qPCR.

Pericardial macrophages, cardiac macrophages, and monocytes pooled from bone marrow, blood, and spleen were sorted for downstream qPCR analysis. Pericardial macrophages were sorted as viable CD45^+^CD11b^+^CD102^+^. Heart lysates were first enriched with CD45 MACS beads (Miltenyi Biotec), and cardiac macrophages were sorted as viable CD45^+^CD11b^+^CD64^+^Ly6C^lo^. Blood, spleen, and bone marrow cell suspensions were pooled and enriched using a monocyte enrichment kit (Miltenyi Biotec) and were sorted as viable CD45^+^CD11b^+^CD64^lo^Ly6C^hi^SSC^lo^.

Upon collection, cells were lysed in 350 μL Buffer RLT (QIAGEN), and RNA was subsequently isolated using the RNeasy Plus Micro Kit (QIAGEN). Using 1 μg of isolated RNA per sample, cDNA was produced with the High-Capacity cDNA Reverse Transcription Kit (Applied Biosystems). *Cst3* gene expression levels (forward: 5′-CTTTCCATGACCAGCCCCAT-3′; reverse: 5′-GCACCCTTCTGCGAGATGAA-3′; Integrated DNA Technologies) were then determined by quantitative reverse transcription PCR (RT-qPCR). Using SYBR Green PCR Master Mix (Applied Biosystems) with 5 ng of cDNA per reaction, RT-qPCR reactions were completed in a 96-well PCR plate (half skirt, low profile, transparent) (Sarstedt), overlaid with an Optical Adhesive Cover (Applied Biosystems) in the CFX96 Touch Real-Time PCR Detection System (Bio-Rad). PCR data were assessed using the ΔΔCt method relative to 18S ribosomal RNA (Quantitect) as a housekeeping gene, and analyzed using Prism (GraphPad, version 10.1.0). Procedures were completed per manufacturer protocols.

### Cystatin C concentration.

Human PFcs (total and enriched populations) were collected from 8 NOCAD and 8 MI patients and cultured in media. Cystatin C concentration was measured with blinded multiplex analysis (Eve Technologies). Circulating and pericardial lavage mouse cystatin C concentrations were measured in *Cst3^ΔLyz2^* and *Cst3^fl/fl^* littermates by ELISA (Abcam).

### Statistics.

Patient characteristics are expressed as mean ± SD or count (percentage) where appropriate. Statistical analysis was conducted using GraphPad Prism 10 software. We considered *P* values less than 0.05 as significant.

### Study approval.

The Conjoint Health Research Ethics Board at the University of Calgary approved the protocols of all human studies (ethics ID: REB16-1906; approved February 12, 2021), and each investigation was performed in accordance with the Declaration of Helsinki. All study participants provided written informed consent prior to participation.

All animal experiments were conducted in compliance with the animal protocol approved by the University of Calgary Animal Care Committee and were in accordance with the guidelines defined by the Canadian Council on Animal Care.

### Data availability statement.

The authors confirm that the data presented herein are contained within the article and its supplemental materials. Single-cell RNA-seq datasets are available via the NCBI’s Gene Expression Omnibus (GEO) with accession number GSE188890. Single-cell datasets can be further explored via our companion portal at www.biernaskielab.ca/Pericardial_Fluid or www.biernaskielab.com/Pericardial_Fluid, which were generated using the RShiny (v1.1.0), shinylp (v1.1.2), and shinythemes (v1.1.2) packages. Velocyto-generated loom files and processed R objects are available for reanalysis from https://doi.org/10.6084/m9.figshare.33265461 Values for all data points in graphs are reported in the Supporting Data Values file.

## Author contributions

AFH and JFD designed the research studies. AFH, SS, DB, NLR, EL, DY, FIS, BHL, JAD, CAI, AT, HBS, ANZ, EG, JAB, and JFD conducted the experiments and acquired data. JDT collected patient samples and conducted experiments. SS, AJ, DY, KC, JAD, EDC, AT, A Derakhshani, EG, and JFD performed the analysis. SSO provided *Cst3*-floxed mice. AFH, PWMF, and JFD wrote the manuscript with input from all coauthors. All authors read and approved the manuscript for submission. A Dufour, JAB, PWMF, and JFD supervised the study.

## Conflict of interest

The authors have declared that no conflict of interest exists.

## Funding support

The following organizations provided financial support:

- Canadian Institutes of Health Research (Project Grant) (to JFD and PWMF).
- Canadian Institutes of Health Research (Vanier Canada Graduate Scholarship) (to AFH).
- Killam Foundation (Doctoral Award) (to AFH).
- Alberta Innovates: Health Solutions (Doctoral Scholarship) (to AFH).
- Kertland Family Doctoral Award (to AFH).
- German Academic Exchange Service (Biomedical Education Program) (to FIS).
- Canadian Institutes of Health Research (Canada Graduate Scholarship–Doctoral) (to JAD and CAI).
- Canadian Institutes of Health Research (Canada Graduate Scholarship–Masters) (to EDC).
- Natural Sciences and Engineering Research Council of Canada (Vanier Canada Graduate Scholarship) (to HBS).
- O’Brien Centre Summer Studentship Award (to EG).

## Supplementary Material

Supplemental data

Supporting data values

## Version 1 07/16/2026

In-Press Preview

## Version 2 09/08/2026

Electronic publication
