# Supplementary material for: Human pericardial macrophages suppress cardiac fibrosis through cystatin C signaling after myocardial infarction
Source: JCI Insight. 2026 Jul 16;11(17):e202383. doi: 10.1172/jci.insight.202383 (PMC13564072; doi:10.1172/jci.insight.202383)
Supplement: Supplementary file 1 [file jciinsight-11-202383-s147.pdf]

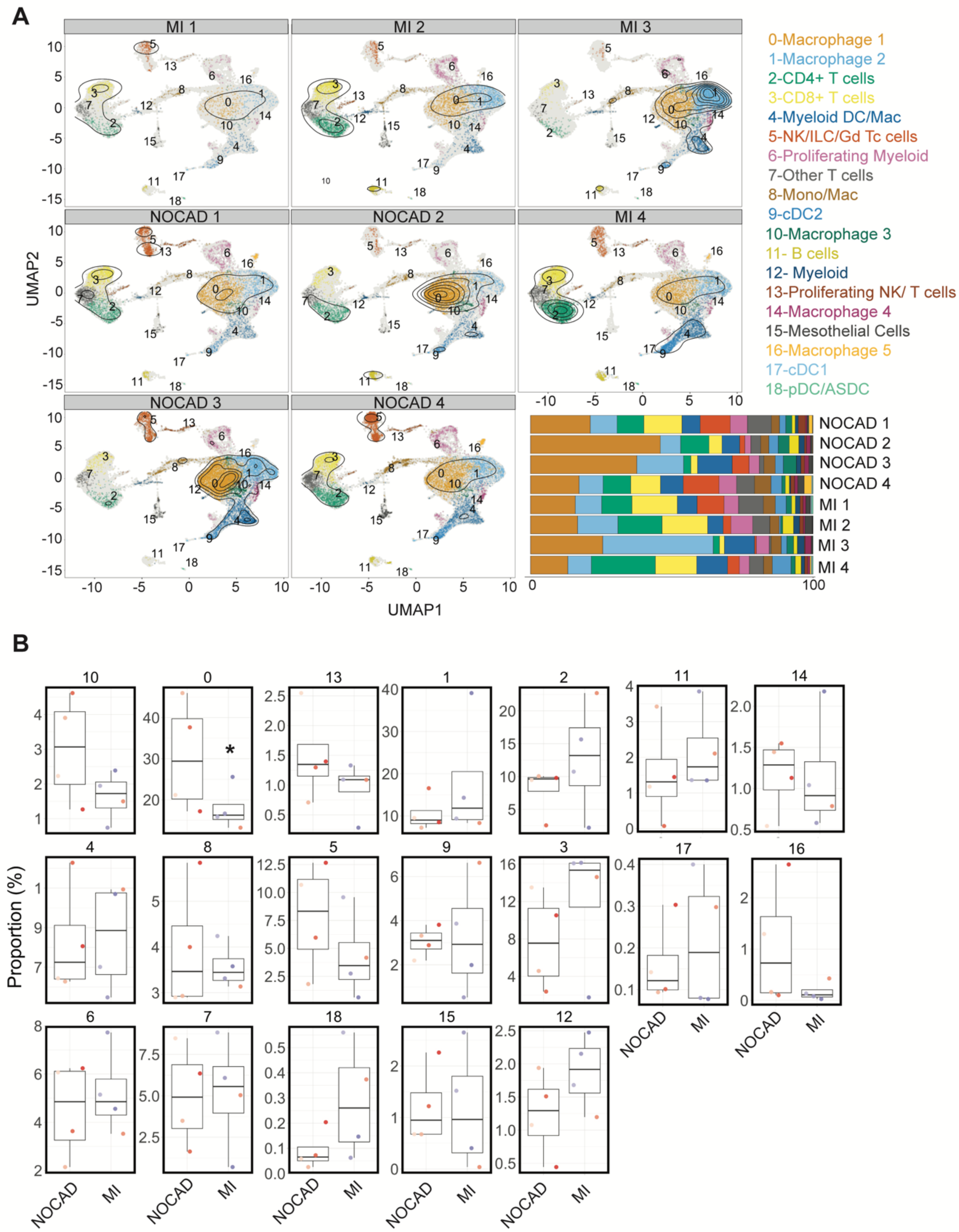

Supplemental Figure 1: Patient-based pericardial fluid cell clusters.

A, UMAP projections and stacked plot representation of 19 distinct pericardial fluid cell population for 8 individual patients. B**,** Boxplots showing percentage of each cell cluster grouped by clinical cohort. n=4 for NOCAD and n=4 for MI patients. *=p<0.05 using a generalized linear mixed-effects model.

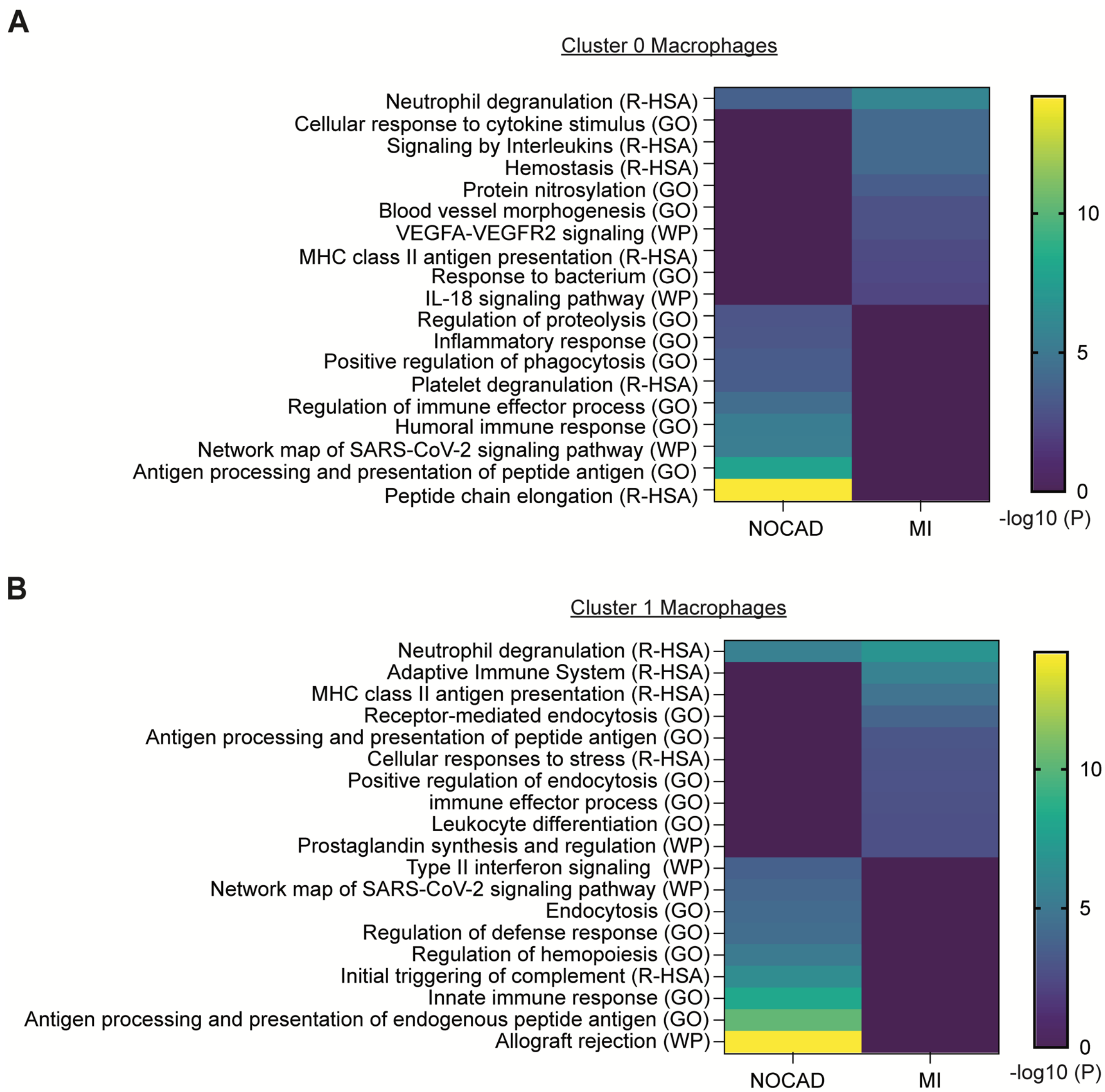

Supplemental Figure 2: Myocardial injury shifts pericardial macrophage phenotype.
Metascape pathway analysis of DEGs in both Cluster 0 and Cluster 1 pericardial macrophages between NOCAD and MI samples. The DEGs were calculated with Seurat (v.4) Wilcoxon rank-sum test, which had an average logFC greater than 0.25 and a p-adjusted value of less than 0.05. The cumulative logFCs were generated for each Louvain cluster to indicate the discrepant genes on a cluster-by-cluster basis.

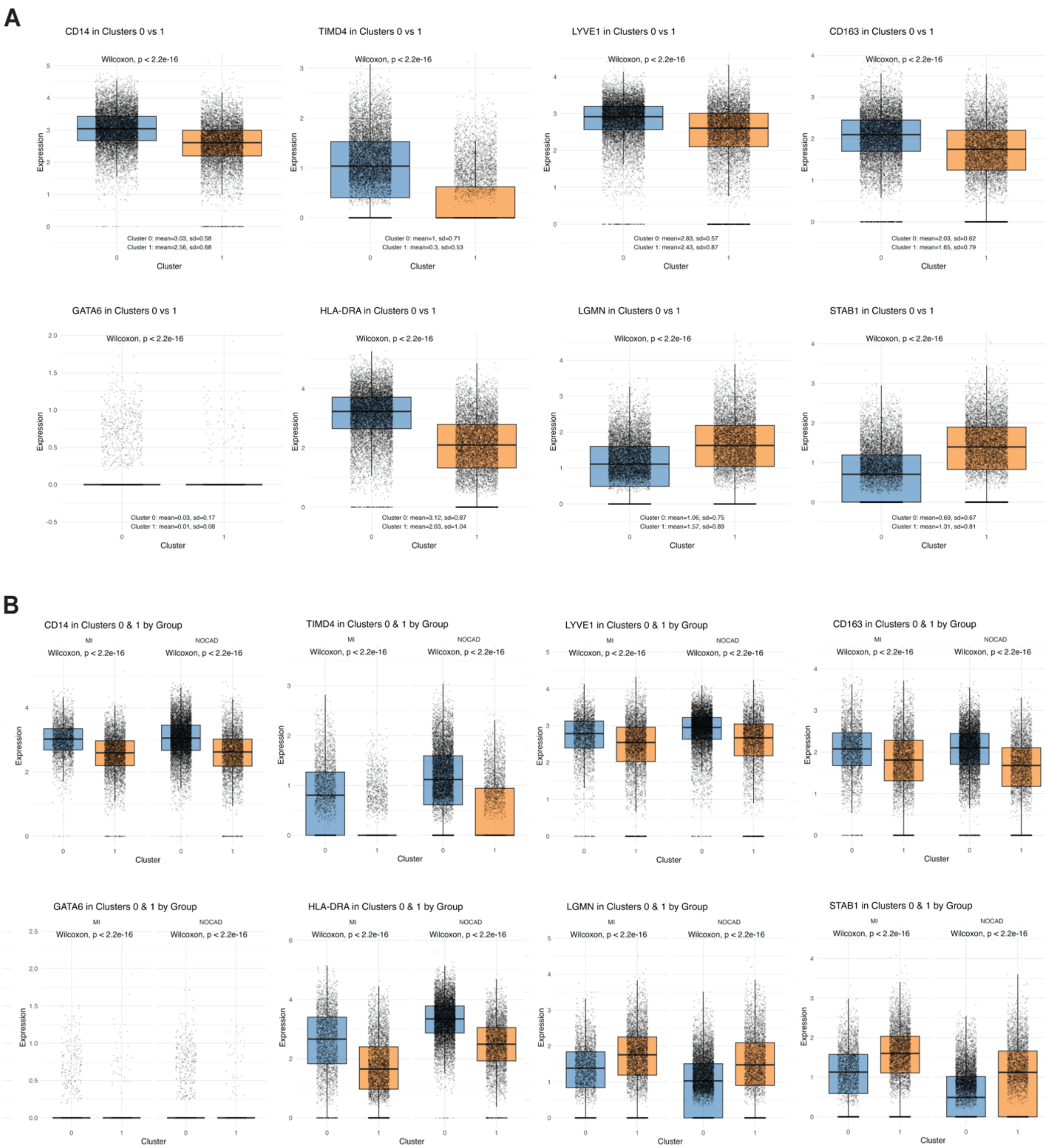

Supplemental Figure 3: Gene marker expression for cluster 0 and 1 pericardial macrophage populations.

A-B, Boxplots for defining genes (CD14, TIMD4, LYVE1, GATA6, HLA-DRA, CD163, LGMN, STAB1) comparing expression across clusters (0 vs. 1) overall (A) and separated by patient groups (MI vs. NOCAD) (B). Each plot includes mean, standard deviation, and Wilcoxon p-values.

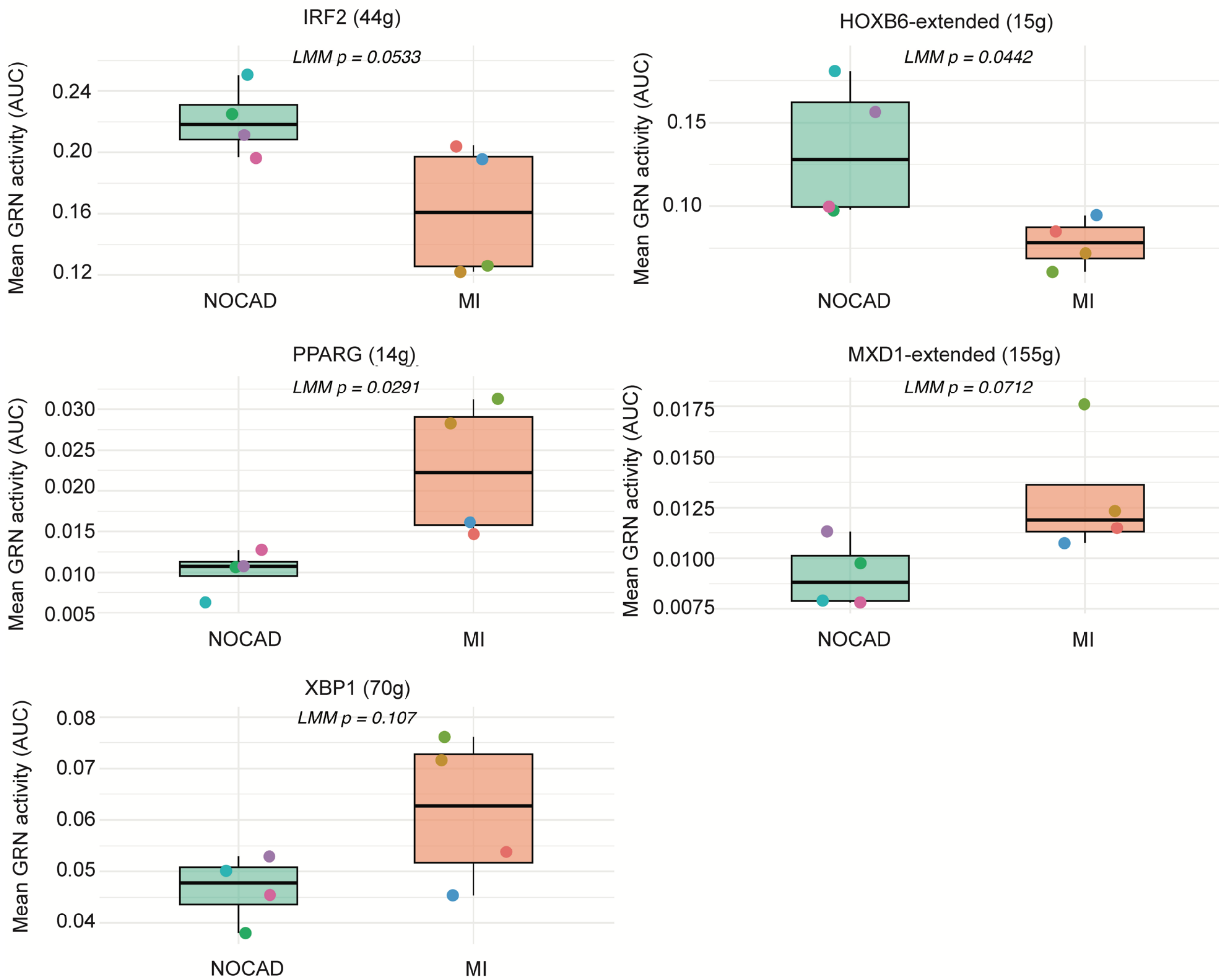

Supplemental Figure 4: Gene regulatory network clustering analysis for pericardial macrophages for individual patients.

Analysis of gene regulatory networks for IRF2, HOXB6, PPARG, MXD1, XBP1 transcription factors.

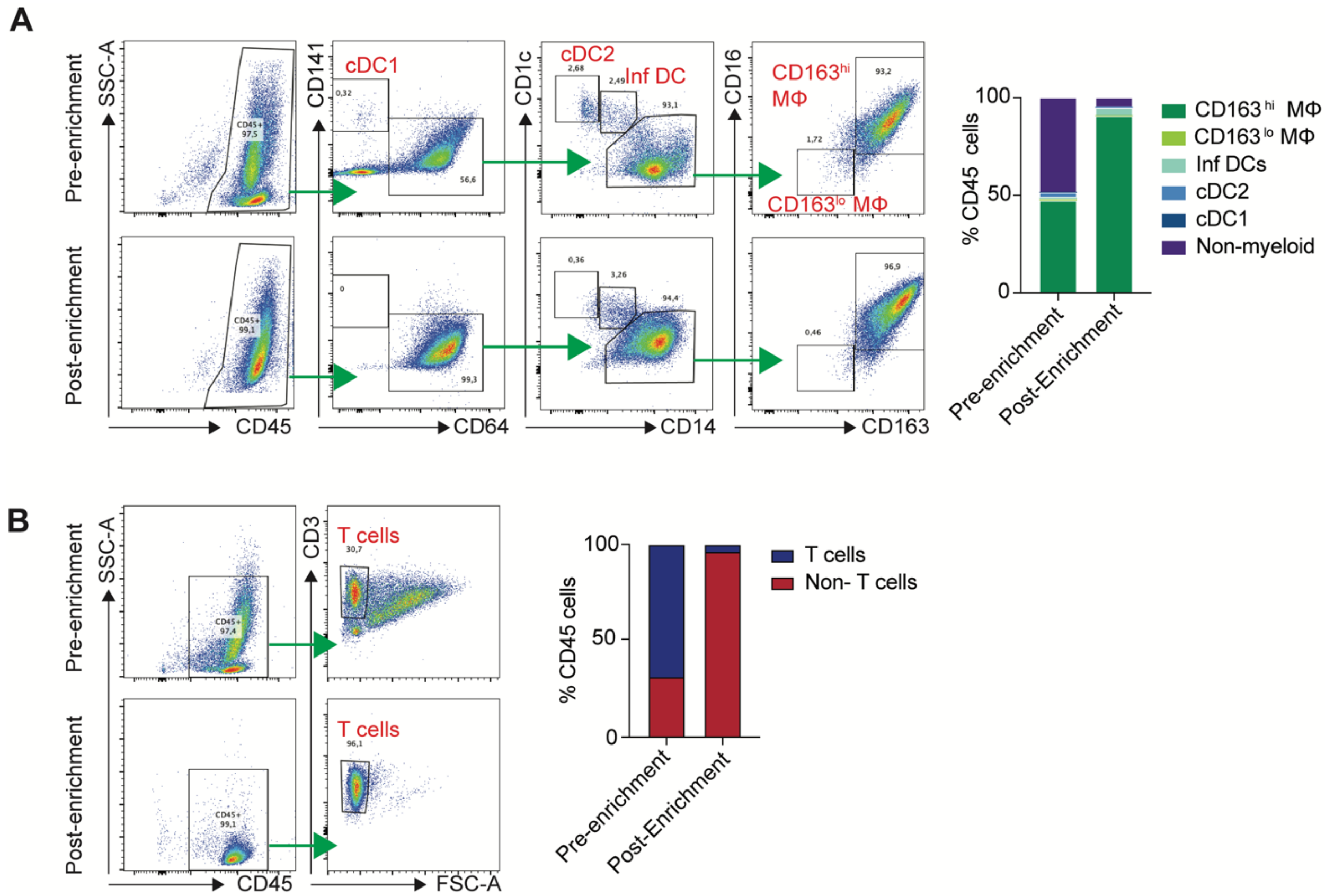

Supplemental Figure 5: Magnetic bead enrichment for pericardial macrophages and T cells.
A, Representative flow plots and quantification of pericardial myeloid populations with or without CD14 bead enrichment. B, Representative flow plots and quantification of pericardial T cells s with or without CD3 bead enrichment. Representative of two independent experiments.

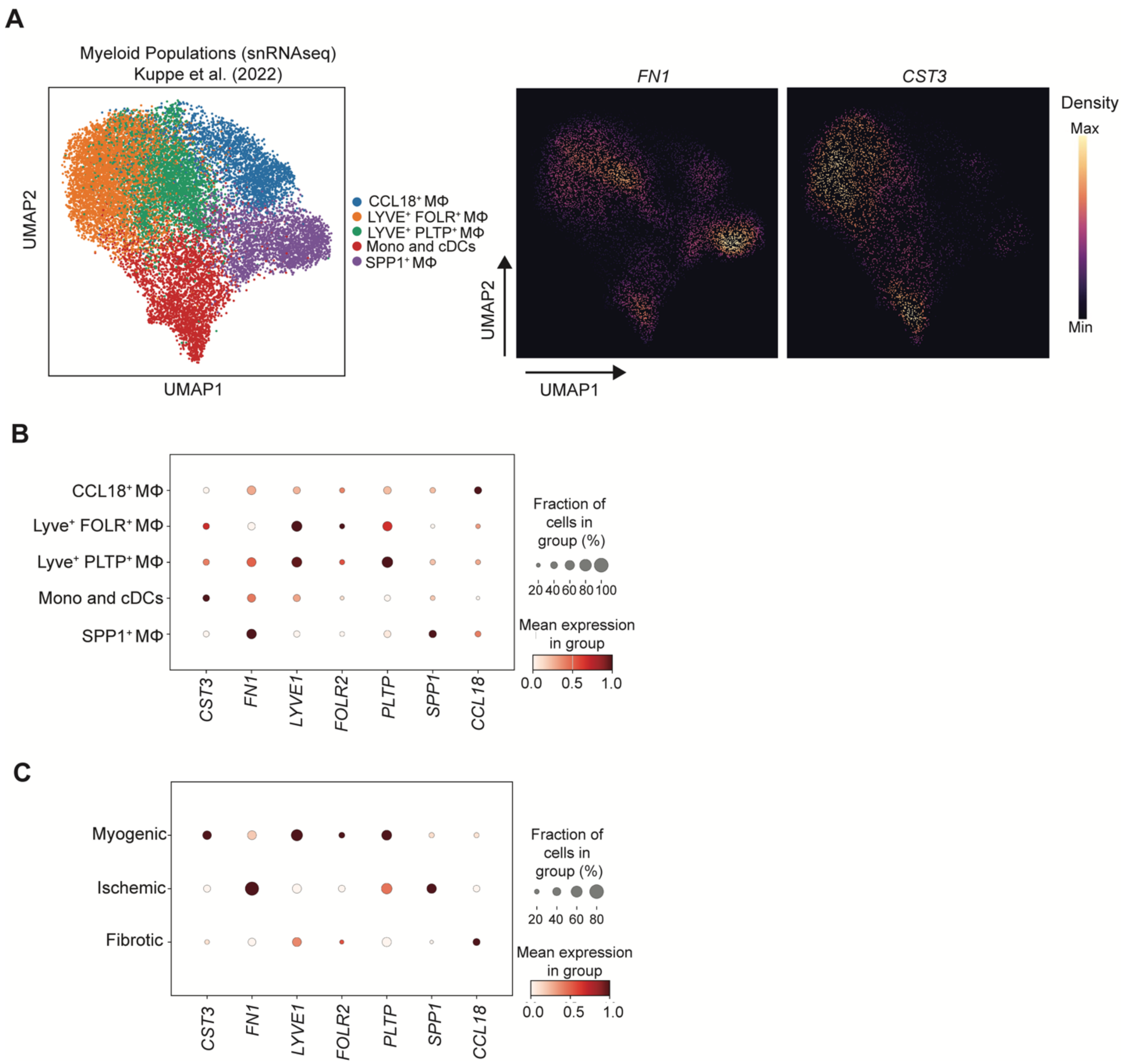

Supplemental Figure 6: FN1 and CST3 cardiac macrophage expression following ischemic injury.
A, Left: Annotated myeloid population UMAP of cardiac macrophage clusters from snRNAseq dataset (21). Center & Right: KDE density maps for FN1 (concentrated in Monocytes/cDCs cluster, lower right) and CST3 (broad monocyte/macrophage expression). B, Dot plot of marker gene expression across named myeloid populations. LYVE1 and PLTP highly expressed in LYVE+ macrophages; CST3 marks Monocytes/cDCs; SPP1 marks SPP1+ MQs; CCL18 marks CCL18+ MQs. Dot size = % cells expressing; color = scaled mean expression. C, Dot plot of marker gene expression across 3 patient disease groups; myogenic, ischaemic, and fibrotic.

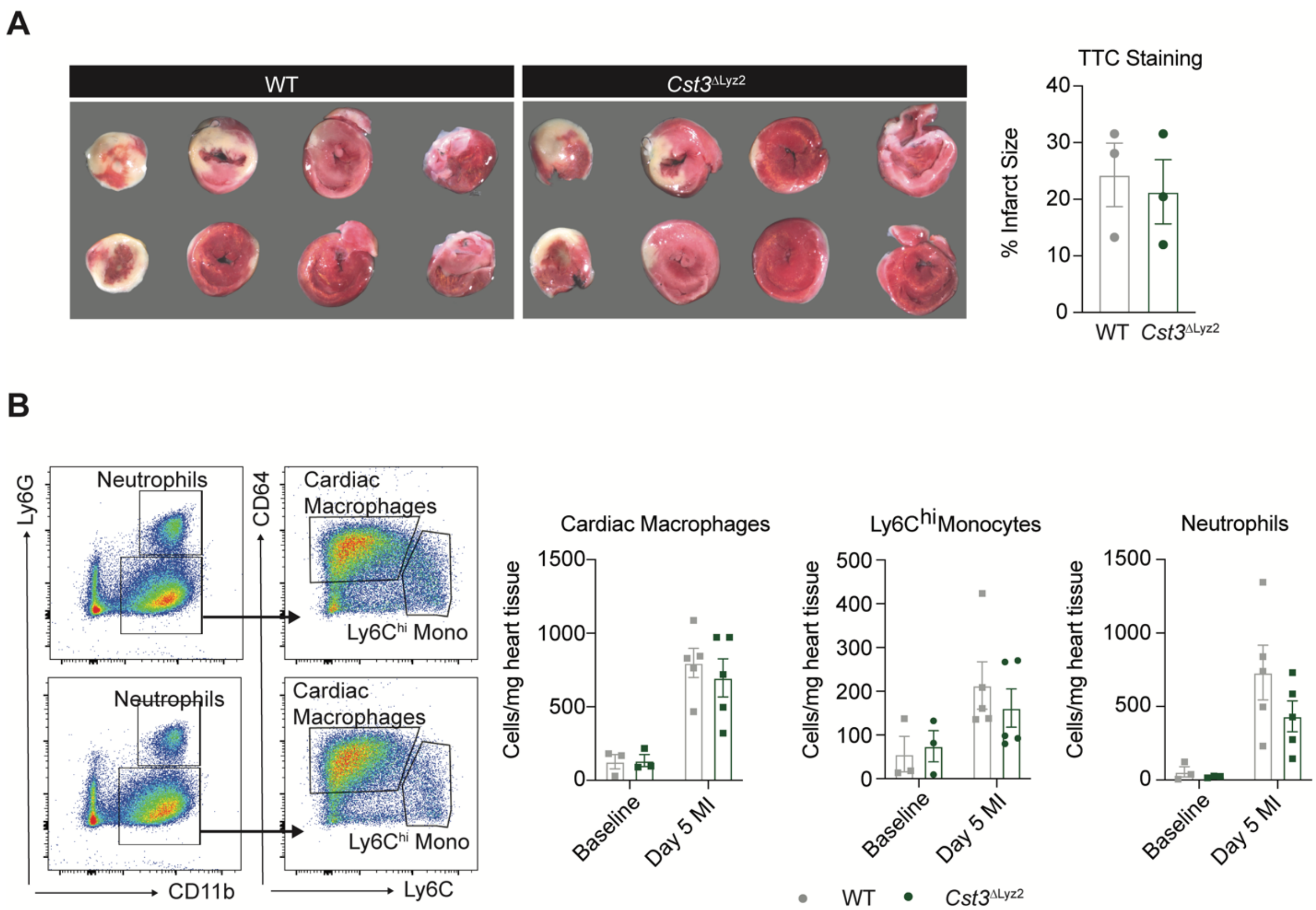

Supplemental Figure 7: Infarct size and myeloid cell recruitment in myeloid Cst3 deficient mice. A, Representative TTC staining and quantification of infarct size (necrotic area/total LV area) at 48 hrs post-MI in WT and $Cst3^{\Delta Lyz2}$ mice. n =3/group. B, Representative flow plots and quantification of neutrophil, $Ly6C^{hi}$ monocytes, and cardiac macrophages at baseline and 5 post-MI in WT and $Cst3^{\Delta Lyz2}$ mice. n=3/group for baseline samples and n=5/group for day 5 post-MI samples.

Variable	MI (n=4)	NOCAD (n=4)
Mean Age ± SD (years)	63.5 ± 5.4	60.0 ± 7.0
Gender		
Male	4 (100)	4 (100)
Hypertension	4 (100)	0 (0)
Dyslipidemia	3 (75)	0 (0)
Diabetes		
Type I	0 (0)	0 (0)
Type II	1 (25)	0 (0)
Insulin-dependent	0 (0)	0 (0)
Smoking		
Active	0 (0)	0 (0)
Remote	0 (0)	0 (0)
Atrial Fibrillation		
Paroxysmal	1 (25)	1 (25)
Permanent	0 (0)	0 (0)
Severe LV dysfunction	0 (0)	0 (0)
Chronic kidney disease		
eGFR <45 mL/min	0 (0)	0 (0)
Dialysis dependent	0 (0)	0 (0)
Chronic obstructive lung disease	0 (0)	0 (0)
Peripheral arterial disease	0 (0)	0 (0)
Cerebrovascular disease	0 (0)	0 (0)
Rheumatoid disease	0 (0)	0 (0)
Autoimmune disease	0 (0)	0 (0)
Immunosuppressed	0 (0)	0 (0)
Active infective endocarditis	0 (0)	0 (0)
Preoperative medication use		
Steroids	0 (0)	0 (0)
Aspirin	0 (0)	0 (0)
NSAIDs	0 (0)	0 (0)
Anti-inflammatory	0 (0)	0 (0)
Antibiotics	0 (0)	0 (0)

Supplementary Table 2. Baseline patient demographics for single cell RNA sequencing of pericardial fluid. MI- myocardial infarction**,** CAD- coronary artery disease, LV- left ventricular, eGFR- estimated glomerular filtration rate, NSAIDs- nonsteroidal anti-inflammatory drugs. Represented as patient number with the % in parentheses.

Cluster 0	Cluster 1	Cluster 2	Cluster 3	Cluster 4	Cluster 5	Cluster 6	Cluster 7	Cluster 8	Cluster 9	Cluster 10	Cluster 11	Cluster 12
FN1	DAB2	LTB	GZMK	VCAN	GNLY	TOP2A	IL7R	FTL	HLA-DPB1	MT1G	IGKC	NEAT1
IFI27	CCL2	MAL	CCL5	LYZ	NKG7	MKI67	IL32	BLVRB	FCER1A	MT2A	IGHM	MALAT1
C1QB	FTL	LDHB	IL32	FCN1	KLRD1	STMN1	KLRB1	IFI27	HLA-DPA1	MT1X	IGLC2	MT-ND3
CFD	RNASE1	CD3D	CD2	HLA-DRA	TRDC	CENPF	LTB	PRDX1	HLA-DQA1	SOD2	IGLC3	KCNQ1OT1
LYPD2	GLUL	IL7R	CST7	HLA-DRB1	CCL5	H2AFZ	CD52	RNASE1	HLA-DRA	FN1	JCHAIN	MT-CO3
HAMP	LGMN	CD3G	CD8A	HLA-DPA1	XCL2	TUBA1B	CD2	C1QC	HLA-DRB1	CCL4	CD79A	MT-CYB
C1QC	CTSB	RPS3	GZMA	MNDA	GZMA	HMGN2	CD40LG	CSTB	HLA-DQB1	CCL3	MS4A1	MT-ATP6
C1QA	F13A1	CCR7	CD8B	HLA-DRB5	IL2RB	HIST1H4C	CD3D	LGALS3	CD1C	RHOB	IGHA1	MT-ND5
MARCO	NEAT1	CD3E	GZMH	HLA-DPB1	KLRC1	TUBB	CD3G	SELENOP	HLA-DRB5	NAMPT	VPREB3	VMP1
FCGR3A	BRI3	CD52	CD3D	MARCKS	KLRB1	NUSAP1	TRAC	SLC40A1	CLEC10A	LMNA	RALGPS2	MT-CO2
PSAP	CALM3	RPL34	TRAC	CD74	CTSW	HMGB2	SOCS1	NUPR1	CD74	IFI27	IGHD	MT-CO1
C2	FABP5	RPL10	CD3G	CD36	XCL1	ASPM	CD3E	S100A9	HLA-DQA2	FTH1	CD79B	MT-ND1
LYVE1	BLVRB	RPL3	TRBC2	FGL2	CLIC3	PTTG1	TRBC2	S100A8	CPVL	CXCL3	BANK1	CD163
FTH1	TTYH3	RPS27	CD3E	CLEC10A	CMC1	PCLAF	BCL2	C1QB	CST3	SAT1	CD37	N4BP2L2
CYP27A1	STAB1	RPL32	NKG7	HLA-DQB1	STK17A	CDK1	SYNE2	FTH1	LGALS2	CXCL10	IGHG3	MT-ND2
TIMP1	CSTB	RPS8	GZMM	MEF2C	CD7	TYMS	ETS1	BRI3	HLA-DMA	LYPD2	TCL1A	POLR2J3.1
PLTP	S100A8	RPL18	CD52	AP1S2	CCL4	TUBA1C	CISH	PEPD	IGSF6	NCF1	IGHG1	WSB1
CD14	CTSD	RPS12	TRBC1	S100A12	MATK	TPX2	RORA	ENHO	LYZ	CFD	BIRC3	MT-ND4L
CYP1B1	LGALS1	RPL30	CCL4	AC020656.	CST7	CEP55	TRBC1	FCGRT	CD1E	MT1E	LINC00926	MT-ND4
FOLR2	ADAP2	RPS18	HCST	RNF141	HOPX	CDKN3	INPP4B	AIF1	JAML	NUPR1	MZB1	SLC11A1
CST3	NPL	TRAC	APOBEC3G	HLA-DQA1	GZMM	BIRC5	SPOCK2	S100A11	FGL2	C5AR1	CD52	RSRP1
CD163	ANXA4	RPS15A	KLRG1	ZEB2	HCST	KPNA2	DPP4	C1QA	BASP1	EMP1	BCL11A	MT-ND6
VSIG4	IDH1	TCF7	RARRES3	CD93	PRF1	CKS1B	CXCR4	TSPO	CKLF	ACSL1	CXCR4	DDX17
MS4A7	SPP1	RPSA	ETS1	SERPINA1	APOBEC3G	SMC4	RCAN3	FOLR2	GAPT	TNFAIP2	HVCN1	ZEB2
ALDH2	CTSZ	TRBC2	IKZF3	KCTD12	GSTP1	TK1	MYL12A	CLTA	LSP1	NFKBIA	LINC02397	DDX5

Supplemental Table 2. Top cluster markers.

Cluster 13	Cluster 14	Cluster 15	Cluster 16	Cluster 17	Cluster 18
STMN1	FCGBP	ITLN1	FCGBP	CPVL	GZMB
HIST1H4C	APOE	SLPI	CD93	HLA-DPB1	JCHAIN
HMGB2	SPP1	C3	MSR1	LGALS2	PTGDS
TOP2A	APOC1	CCDC80	SLC40A1	HLA-DPA1	CCDC50
MKI67	A2M	PRG4	CCL2	DNASE1L3	IRF8
CENPF	FSCN1	PLA2G2A	SELENOP	HLA-DQA1	TCF4
NUSAP1	LGALS1	KRT19	FCGR2A	CPNE3	UGCG
TYMS	TREM2	MGP	HPGDS	S100B	IRF7
HIST1H1D	DAB2	KRT18	STAB1	CLEC9A	BCL11A
GNLY	CTSL	HP	BCAT1	C1orf54	CLIC3
ASPM	C1QC	RARRES2	CTSD	IRF8	PPP1R14B
HMGN2	CYFIP1	ADIRF	MAMDC2	HLA-DRA	C12orf75
DUT	STAB1	TM4SF1	DAB2	HLA-DQB1	PLD4
HIST1H1B	EPB41L2	CALD1	CD28	CD74	IL3RA
H2AFZ	SCIN	TPM1	MRC1	HLA-DRB1	LILRA4
GZMA	IGSF6	CFH	SYNE1	SNX3	ITM2C
SMC4	MARCKS	KRT8	LGMN	HLA-DRB5	MZB1
NUCB2	C1QB	IGFBP5	COLEC12	CST3	TCL1A
TMPO	CD63	C1R	MAF	WDFY4	SPIB
TPX2	HSPA5	CPE	TGFBI	FGL2	APP
PCLAF	DBI	TIMP1	IGSF21	RGS10	SERPINF1
HMGB1	TUBA1B	RARRES1	HLA-DQB1	XCR1	PLAC8
ATAD2	CTSB	SERPING1	CD74	IDO1	HERPUD1
HIST1H1C	CD14	MSLN	PTMS	BATF3	DERL3
ANP32E	GSN	C1S	ADAP2	RGCC	TPM2

Supplemental Table 2. Top cluster markers. (continued)

Cluster 0

Gene	A477	A490	A500	A575	Sum
CCL18	0	0.75499651	3.22452625	0.26500975	4.2445325
LGALS1	0.69092791	1.22920947	1.22551181	0.46679555	3.61244475
STAB1	0.61080335	1.60861008	0.83299535	0.30608163	3.35849041
CCL2	0.51501321	1.17167261	0.86846111	0.32378168	2.8789286
PPDPF	0.71485979	0.42866017	0.84252226	0.42385638	2.4098986
RNASE1	0.33059997	0.96660726	0	0.8561644	2.15337163
DAB2	0	0.82035038	0.67700631	0.63381583	2.13117252
P4HB	0.53866327	1.05578037	0.42852222	0	2.02296586
CTSB	0.33962653	0.89362386	0.73479954	0	1.96804994
TUBA1A	0.51716644	0.66131812	0.71903554	0	1.8975201
CD55	0.55865892	0	0.87188127	0.45094158	1.88148177
LGMN	0	0.96562652	0.31049409	0.55105296	1.82717357
UCP2	0.66676372	0.75049519	0.34207708	0	1.75933598
HSPB1	0.72483052	0	0.43947266	0.56575189	1.73005508
TAGLN2	0.78925934	0.48329363	0.4221858	0	1.69473877
GAPDH	0.47651757	0.64020041	0.53452839	0	1.65124637
ZYX	0.7362331	0.45883769	0	0.4205836	1.6156544
AP2A2	0.32288468	0.68765228	0.53736092	0	1.54789788
AP2S1	0.4204483	0.40644576	0.44834107	0.27196477	1.5471999
CD99	0.32021692	0.46496597	0.42199243	0.33345779	1.54063311
CREG1	0.35659456	0.50424878	0.25266914	0.35599247	1.46950495
SERF2	0.27454595	0.53692545	0.64689246	0	1.45836385
GLIPR2	0.48251095	0.47112575	0.44484241	0	1.39847911
CFL1	0.51531334	0.50640356	0.34854322	0	1.37026012
SH3BGRL3	0.67880646	0.34957877	0.33944288	0	1.36782811
ECM1	0.61190735	0.25989172	0.42236262	0	1.29416169
ENHO	0.86907921	-0.3246207	-0.2637037	0.96877765	1.24953247
UQCR11	0.36333867	0.48048194	0.38569811	0	1.22951871
LMNA	0.3555613	0.37160363	0.4943469	0	1.22151183
F13A1	-0.61473	0.97042241	0.54698801	0.25287445	1.15555488
ANXA2	0.34121917	0.40813564	0.34497833	0	1.09433314
SPI1	0.4938576	0.34731335	0.25240007	0	1.09357102
TGFBI	0.33028725	0.48072441	0	0.26801714	1.0790288
S100A8	0.29463584	0.45701421	1.14226477	-0.8162557	1.07765917
CSNK2B	0.45236616	0.34855007	0.25594128	0	1.05685751
ENO1	0.35311426	0.38558942	0.28330284	0	1.02200652
POLR2J3.1	0.38517334	0	0.2778982	0.3559967	1.01906824
STXBP2	0.35307171	0.37771496	0.28372307	0	1.01450973

Supplemental Table 3. Cluster perturbation scores.

GAS7	0.29412096	0.3195294	0.39239621	0	1.00604658
RHOG	0.31078555	0.32942049	0.35930067	0	0.99950671
PLEKHO1	0.2890513	0	0.29594454	0.37535357	0.9603494
COX5A	0.26450874	0.34150311	0.3100647	0	0.91607655
FPR1	0	0.63517988	0.52682724	-0.2834883	0.87851883
VIM	0.26371824	0.27557037	0.33065869	0	0.8699473
RHOB	0.74533827	-0.3407004	0	0.27095814	0.67559606
FCER1G	0.43450422	0.51516086	0	-0.3384777	0.61118742
CD52	0.25051797	0.52546493	-0.2886265	0	0.48735637
LAIR1	0.25973056	0.40727817	0	-0.2665506	0.40045814
FOS	1.44828907	-0.4236038	-0.3337795	-0.3205912	0.37031449
ACADVL	0.26213301	0.35013016	0	-0.2522494	0.36001377
PABPC1	0.25331334	0	-0.2721922	0.30873679	0.28985794
KLF2	0.40527813	-0.5396749	0	0.42168772	0.28729095
S100A9	0	0.28878873	0.57258668	-0.6710164	0.19035898
TYMP	0.40198779	0	0.33368528	-0.5570133	0.17865982
JUNB	0.68807669	-0.3147964	-0.2777316	0	0.09554871
RPS26	0	0.44537416	0.35737258	-0.8908981	-0.0881513
HLA-DRB5	1.18278263	-0.4851469	-1.7416946	0.83195613	-0.2121028
NAMPT	0	-0.3496706	0.71732825	-0.59554	-0.2278823
RPS3	-0.2504127	0	-0.2655949	0.26114176	-0.2548659
RPL5	-0.308121	0	-0.3615105	0.37492738	-0.2947041
ADORA3	-0.2587564	0.31752849	-0.3650967	0	-0.3063246
ZFP36	0.32994481	-0.373633	-0.2765492	0	-0.3202375
MDM2	0	-0.2616445	-0.3530292	0.26535836	-0.3493154
ANXA5	0	-0.3290999	0.25288916	-0.2741884	-0.3503991
MX1	0.28919887	-0.4603729	0	-0.2684278	-0.4396019
RPL7A	-0.3903813	0	-0.3650107	0.31490069	-0.4404913
CD300E	0	-0.4053285	0.33648241	-0.3762748	-0.4451209
DUSP1	0.51164508	-0.5544721	0	-0.4232959	-0.4661229
EPB41L3	-0.2899147	-0.2885276	0.36039696	-0.3285187	-0.546564
RPS27L	-0.3056422	0	-0.4967821	0.25061266	-0.5518116
ALDH1A1	0	-0.2800267	-0.5946704	0.31386105	-0.5608361
CFP	0.27297107	-0.5342425	-0.3127628	0	-0.5740342
GAS6	-0.3405074	-0.2511855	-0.3739729	0.34826982	-0.6173959
MAF	0	-0.3024059	-0.7620671	0.40656229	-0.6579108
EEF1A1	0	-0.308719	-0.6525469	0.27098856	-0.6902773
SELENOP	0	-0.3063162	-0.7799963	0.37248443	-0.7138281
FOXC1	0	-0.2948866	-0.2545618	-0.2542582	-0.8037066
LINC02432	-0.3138709	-0.3017403	-0.3018502	0	-0.9174614
UBE2J1	0	-0.3491502	-0.3067884	-0.2733857	-0.9293243

RPS11	-0.2802425	-0.4127932	-0.2869475	0	-0.9799831
SARAF	-0.2778694	-0.2967466	-0.4153568	0	-0.9899727
SLC1A3	-0.3469748	0	-0.265371	-0.3810387	-0.9933845
PDK4	0.6037706	-0.2581704	-0.542195	-0.7996568	-0.9962516
ERAP2	-0.4518304	-0.5646561	-0.2894806	0.29622329	-1.0097439
AC020656.1	-0.3608265	-0.3296509	-0.3403229	0	-1.0308004
RPL30	-0.3785726	-0.3206521	-0.3414922	0	-1.040717
A2M	0	-0.5398949	-1.0231425	0.50565414	-1.0573832
EPS8	-0.2802989	-0.333191	-0.4439308	0	-1.0574208
PDE4DIP	-0.4794165	-0.2957211	0	-0.3237596	-1.0988971
APOE	0	-0.3856421	-0.389795	-0.3385987	-1.1140357
CLIC4	-0.286332	-0.4501381	-0.3886367	0	-1.1251068
KCNMA1	-0.556046	0	-0.2599353	-0.3398803	-1.1558616
APOC1	0	-0.3343791	-0.4692888	-0.3530368	-1.1567047
RPL14	-0.4563711	-0.2657923	-0.4561149	0	-1.1782783
RPS27	-0.491679	-0.3893219	-0.2975761	0	-1.1785771
TCF4	-0.340059	-0.3425072	-0.5254254	0	-1.2079917
DENND3	-0.3802865	-0.3121785	-0.517271	0	-1.209736
HLA-F	0	-0.4491276	-0.4642353	-0.3077897	-1.2211525
RPL21	-0.2750024	-0.3719088	-0.5774727	0	-1.2243839
MS4A7	0	-0.4291558	-0.4377607	-0.3671659	-1.2340824
ALOX5AP	-0.6658544	0.69786507	-1.2781776	0	-1.246167
CLEC7A	0	-0.2982036	-0.6687475	-0.2882816	-1.2552327
GPR65	0	-0.3102842	-0.4979923	-0.4642589	-1.2725354
MSLN	-0.3072893	-0.3302913	-0.3271803	-0.3253724	-1.2901334
GATM	-0.3593079	-0.3794015	-0.5514631	0	-1.2901726
TPT1	-0.2931703	-0.4609848	-0.5522191	0	-1.3063743
LTA4H	0.39367655	-0.6459725	-0.5566717	-0.5031204	-1.3120881
HLA-C	0.35841155	-0.3574542	-0.7157236	-0.6020382	-1.3168044
MT-ND2	-0.5113568	-0.2921838	-0.5423051	0	-1.3458456
RND3	0	-0.4512664	-0.4766929	-0.4243655	-1.3523248
FCGR2B	-0.5890194	-0.3330915	-0.4319588	0	-1.3540697
EVI2B	-0.2799989	-0.4936858	-0.6224463	0	-1.396131
RPL23	-0.4225452	-0.5165537	-0.470802	0	-1.4099009
TIMD4	0	-0.4482691	-0.4601465	-0.5414347	-1.4498503
RPL27A	-0.4085112	-0.5138382	-0.6289201	0	-1.5512695
MT2A	0	-0.4079	-0.5538253	-0.599196	-1.5609214
LYPD2	-0.6106739	-0.6850652	-0.2959237	0	-1.5916628
EPB41L2	-0.4837583	-0.5636658	-0.5531435	0	-1.6005676
C1QB	-0.5128335	0	-0.7451312	-0.3731338	-1.6310985
LYZ	-0.4171228	-0.5325242	-0.7221849	0	-1.6718319

CD81	-0.5724961	0	-0.6826052	-0.4624984	-1.7175997
CXCL16	-0.4735833	-0.5386059	-0.4274186	-0.366273	-1.8058809
HLA-DQB1	0	-1.0802091	-0.9934974	0.26726488	-1.8064415
FCGR3A	-0.3218059	-0.6382585	-0.7493938	-0.3018427	-2.0113009
SOD2	-0.5472885	-0.9717433	0	-0.5609159	-2.0799478
BHLHE41	-0.5429083	-0.9155277	-0.643478	0	-2.1019141
FCN1	0.51232207	-1.3353316	-1.0656212	-0.2735317	-2.1621624
PLAC8	0	-0.5379664	-1.3748783	-0.3990653	-2.3119101
ALDH2	-0.701388	-0.8350574	-0.8491526	0	-2.385598
CLEC12A	-0.8030932	-0.8501515	-0.4150238	-0.4631764	-2.531445
IFI27	0	-1.1146084	-0.5459476	-0.9116099	-2.572166
ID3	-0.6164384	-0.457001	-1.0578639	-0.5483141	-2.6796174
HLA-DPB1	0	-1.7590563	-1.4337128	0.38030257	-2.8124665
FN1	0	-0.7140882	-1.4293681	-0.7187337	-2.86219
VMO1	-1.100735	-0.8442467	0	-1.0618363	-3.006818
TSPAN13	-0.8375365	-0.7024168	-0.9761597	-0.9405053	-3.4566182
CST3	-0.5433423	-1.2426392	-1.7584579	0	-3.5444395
NUPR1	-1.3860161	-0.8138788	-0.8314039	-0.7918529	-3.8231516
HLA-DRB1	-0.6688357	-1.4162379	-1.819273	-0.3696553	-4.2740018
CFD	-2.1220831	-2.3250649	-1.351949	-0.4343986	-6.2334956

Cluster 1

Gene	A477	A490	A500	A575	Sum
CCL2	0.86056081	1.46181403	1.13786467	0	3.46023951
PPDPF	0.65673731	0.65323773	1.12984088	0.51301867	2.95283459
FABP5	0.42228247	1.26831764	1.06879762	0	2.75939773
PLTP	0.38637576	1.16736687	1.07116879	0	2.62491143
TUBA1B	0.42751979	1.35866313	0.68649432	0	2.47267724
STAB1	0.58007518	1.06725842	0.36642395	0.37818793	2.39194548
S100A6	0.55147736	0.72048775	1.05359527	0	2.32556038
TAGLN2	0.87028282	0.49222113	0.54744052	0.26591475	2.17585923
CD99	0.34592761	0.53905014	0.60933022	0.51592331	2.01023129
LGALS1	0	0.82175124	0.80477395	0.34691021	1.9734354
GLIPR2	0.74284219	0.61837073	0.60228299	0	1.9634959
TUBA1C	0.47676203	0.81110637	0.62098026	0	1.90884866
DAB2	0	0.60843225	0.63381215	0.64500601	1.88725041
CD36	0.2528062	0.55173122	0.61885334	0.46223816	1.88562893
UCP2	0.53837635	0.928363	0.27075877	0	1.73749812
ZYX	0.75448819	0.42563151	0	0.53371344	1.71383314
FCER1G	0.49119948	0.80755621	0.39975124	0	1.69850692
GAPDH	0.52911629	0.65025466	0.5056665	0	1.68503744
TYROBP	0.34917634	0.41793347	0.54527891	0.30693557	1.61932429
ELOB	0.33298507	0.55443457	0.67870067	0	1.56612031
RAB5C	0.35367784	0.54144147	0.38240413	0.27874884	1.55627228
RNASE1	0.33152494	0.66901979	-0.4757741	0.98089226	1.50566289
ANXA6	0	0.81175773	0.4091996	0.25821174	1.47916908
CALR	0.42729993	0.75716247	0.28862595	0	1.47308835
SH3BGRL3	0.47199449	0.53920242	0.44007461	0	1.45127152
CTSB	0.38311148	0.63115506	0.41101664	0	1.42528318
TTYH3	0.46469058	0.49106806	0	0.45495084	1.41070948
CSNK2B	0.40052062	0.44830481	0.2660361	0.28760134	1.40246286
G0S2	0.40093722	0.47288482	0.52640466	0	1.4002267
LMNA	0.61338224	0.28906206	0.49384871	0	1.396293
CRIP1	0.2736091	0	0.84544585	0.27223872	1.39129368
UQCR11	0.40985849	0.53462948	0.40320936	0	1.34769732
ENHO	0.91673991	-0.5161751	0	0.91957209	1.32013694
PFN1	0.46621789	0.50885562	0	0.33952487	1.31459838
ATP5ME	0	0.62414894	0.35849643	0.32503378	1.30767914
AP2S1	0.3458965	0.50179343	0.41897703	0	1.26666696
VMA21	0.31131398	0.57588554	0.34501631	0	1.23221583
MCOLN1	0.27314984	0.29942536	0	0.65952426	1.23209945

PDIA4	0.31380354	0.61089348	0.27879278	0	1.20348981
ANXA2	0.36179547	0.47464388	0.33596093	0	1.17240028
FOS	1.90456019	-0.422656	-0.3233041	0	1.15860004
AP2A2	0.32766594	0.40492558	0.37355838	0	1.10614989
CD55	0.54831733	-0.4212884	0.61762324	0.35799572	1.10264792
FKBP8	0.44420165	0.35135901	0.27769396	0	1.07325462
HLA-DRB5	1.06015252	0	-1.0128811	1.02476913	1.07204052
COX5A	0.3291393	0.38674295	0.30776735	0	1.0236496
HSBP1	0.29965969	0.38745592	0.33148487	0	1.01860048
MYO1G	0.2545803	0.26040601	0.47183116	0	0.98681746
SEC11C	0.27781039	0.36417918	0.30528566	0	0.94727523
RPS26	0.32935096	0.73496984	0.51689497	-0.6825563	0.89865944
IER2	1.48776251	-0.2980998	0	-0.2929688	0.89669384
GNPDA1	0	0.32949968	0.26680221	0.28870774	0.88500964
HLA-A	0.82404538	0	-0.3291409	0.27199667	0.76690119
FPR1	0	0.61070467	0.44119506	-0.3075642	0.7443355
ANXA4	0	-0.3925105	0.40608043	0.40453657	0.4181065
CTSL	0	0.34786676	0.40490364	-0.3988011	0.35396929
KLF2	0.52339174	-0.4767428	0	0.30253768	0.34918665
INSIG1	0	-0.3137445	0.3401799	0.29215414	0.31858957
S100A8	0	0.51825009	0.92981233	-1.1845721	0.26349033
JUNB	0.80533272	-0.3545871	-0.2992103	0	0.15153529
GRN	0.3431966	0	-0.6224297	0.37251982	0.0932867
MT-ATP6	0.30179977	0	-0.5357055	0.30125435	0.06734864
S100A9	0	0.28801321	0.44346424	-0.8716247	-0.1401472
PSME2	0.68513064	-0.2504068	-0.2670297	-0.3291024	-0.1614082
PDK4	0.96546669	0	-0.4112346	-0.8664408	-0.3122087
AC026369.3	-0.3913766	-0.2728846	0	0.28621002	-0.3780512
ATP1B1	0	-0.3015168	-0.3854316	0.25570257	-0.4312458
MAFB	0.38883234	-0.494986	0	-0.3262061	-0.4323598
IRF1	0.40694196	-0.581527	0	-0.2591423	-0.4337273
MTRNR2L8	0	-0.4262818	-0.3054887	0.26988184	-0.4618886
TYMP	0.36803691	-0.4293545	0.26138999	-0.6631581	-0.4630856
FCN1	0.41687449	-0.4353496	-0.4685864	0	-0.4870615
ZFP36L2	0	-0.3923268	-0.4947281	0.32742173	-0.5596331
PLAC8	0.54972544	0	-0.8288879	-0.2953611	-0.5745236
FTH1	0	-0.6631148	0.50785826	-0.4507835	-0.6060401
MT-CO1	0.33720344	-0.5570987	-0.4242859	0	-0.6441812
MT-CO2	0.25626962	-0.345795	-0.6291155	0	-0.7186409
ALDH1A1	0	-0.5780021	-0.6033181	0.45419804	-0.7271221
MSR1	0.51895631	-0.6031161	-0.7227659	0	-0.8069257

CXCL16	0	-0.2895492	-0.2596915	-0.2822602	-0.8315009
HLA-F	0	-0.2576015	-0.3447438	-0.2650473	-0.8673926
SLC1A3	-0.3618193	0	-0.2525072	-0.2902493	-0.9045758
TGM2	0	-0.3265255	-0.3304605	-0.2854737	-0.9424598
FCHO2	-0.3456209	-0.3430206	-0.2709626	0	-0.959604
B2M	0.26320721	-0.5790023	-0.658293	0	-0.974088
MAF	-0.3706709	-0.4272687	-0.8118029	0.5710349	-1.0387076
STAT1	0	-0.4025326	-0.3685267	-0.2717953	-1.0428546
KCNMA1	-0.3936706	0	-0.3564361	-0.3400561	-1.0901629
NR1D2	-0.3457144	0	-0.4604329	-0.3046874	-1.1108348
TPT1	-0.3045461	-0.2964989	-0.5965367	0	-1.1975817
RPL23	-0.3581303	-0.3352929	-0.5126858	0	-1.2061089
PKIB	-0.3580043	-0.4128939	-0.4452816	0	-1.2161798
GBP1	0	-0.5041953	-0.4388232	-0.2768913	-1.2199098
DENND3	0	-0.3958677	-0.4871826	-0.3542604	-1.2373107
FCGR2B	-0.5600198	-0.3534518	-0.3357745	0	-1.2492461
C2	0	-0.4400375	-0.4935553	-0.3224066	-1.2559994
FCGR3A	0	-0.5334154	-0.4222571	-0.3011468	-1.2568193
LGALS3BP	0	-0.4611932	-0.5354326	-0.2620488	-1.2586746
RPL27A	-0.2575795	-0.3537538	-0.6618344	0	-1.2731678
TIMD4	0	-0.3957564	-0.4844236	-0.4259895	-1.3061696
PLA2G16	-0.4797952	-0.2707675	-0.5863762	0	-1.3369388
ERAP2	-0.4822714	-0.5592872	-0.3236849	0	-1.3652435
SKIL	0	-0.5151157	-0.574832	-0.2801021	-1.3700499
DPYSL2	-0.3325458	-0.3343159	-0.7480191	0	-1.4148807
CXADR	-0.4020033	-0.3040238	-0.4171007	-0.3016295	-1.4247572
HLA-DQB1	0	-0.8521389	-0.8667427	0.28553085	-1.4333507
RND3	0	-0.46144	-0.5404278	-0.459681	-1.4615488
IFI44L	0	-0.5844274	-0.4826411	-0.4092125	-1.4762809
CD81	-0.5201573	0	-0.5483368	-0.44184	-1.5103341
SDC3	0	-0.6990436	-0.5963875	-0.3839821	-1.6794132
TSPAN13	-0.4463598	-0.3612836	-0.4558236	-0.4393427	-1.7028097
HLA-B	0	-0.5174135	-0.6630691	-0.5327085	-1.7131911
MT-ND5	-0.3308219	-0.822689	-0.5878125	0	-1.7413235
IL6ST	0	-0.5229847	-0.7677826	-0.4597148	-1.7504822
HLA-C	0.39596298	-0.8308064	-0.9323633	-0.4685302	-1.835737
CLEC12A	-0.6824759	-0.6265965	0	-0.5404407	-1.849513
ID3	0	-0.4642528	-0.8231501	-0.6182261	-1.905629
SELENOP	-0.8431069	-0.460826	-0.6499209	0	-1.9538538
MPC2	-0.6433976	-0.2817666	-0.7419616	-0.3385431	-2.005669
MT-ND4L	-0.8400544	-0.6216497	-0.573183	0	-2.034887

GATM	-0.5291706	-0.8766442	-0.8693699	0	-2.2751846
HLA-DPA1	0	-1.5772391	-1.1899069	0.28451167	-2.4826343
ALDH2	-0.5026279	-0.771429	-0.7510739	-0.4655768	-2.4907076
HLA-DPB1	0	-1.562679	-1.3079739	0.32117849	-2.5494744
LTA4H	0	-0.9470765	-0.9860536	-0.703033	-2.6361631
CST3	-0.5526625	-1.0704421	-1.0466748	0	-2.6697794
C1QC	-0.4471269	-0.6952483	-1.0338017	-0.5187098	-2.6948867
C1QB	-0.6312409	-0.6378311	-0.8080875	-0.6454402	-2.7225997
HLA-DRA	0	-1.8109757	-1.3726357	0.42462688	-2.7589846
EPB41L2	-0.6250029	-0.9509689	-0.8965795	-0.3647972	-2.8373485
NUPR1	-1.2370814	-0.7545402	-0.7936481	-0.7739205	-3.5591902
HLA-DRB1	-0.6204834	-1.3802143	-1.488412	-0.5028397	-3.9919494
CFD	-1.6529873	-1.7367546	-0.763072	-0.5583076	-4.7111215
IFI27	0	-1.9192096	-1.4082088	-1.6168857	-4.944304

Cluster 2

Gene	A477	A490	A500	A575	Sum
C1QA	-1.0905902	0.5966236	0	-0.7959076	-1.2898742
C1QB	-1.3529228	0.66082043	0	-0.7957255	-1.4878279
CFD	-0.7138873	-0.4824724	0	-0.4669602	-1.6633199
EIF5A	0.58294109	0.4328531	0	0.25353616	1.26933035
FTL	-1.6089687	0	0.29833701	-0.8195067	-2.1301384
MARCO	-0.8399892	0.84501632	0	-0.6239911	-0.6189639
MT-ATP6	0	-0.3507609	-1.1611701	-0.303859	-1.8157899
MT-CO1	0	-0.4658302	-0.4276732	-0.4016949	-1.2951983
MT-CYB	0.31624718	0	-0.4763012	-0.5095286	-0.6695826
MT-ND2	-0.4340937	-0.3625959	-1.3566338	-0.4299687	-2.5832921
MT-ND5	0	-0.5204107	-0.8420484	-0.551253	-1.913712
PSAP	-0.8792205	0.37700492	0.49037307	-0.5115942	-0.5234368
RPL27A	-0.5430844	-0.330175	-0.5276118	0	-1.4008712
S100A8	-1.0873526	0.82453153	1.41304974	-0.7760954	0.37413324
S100A9	-1.4988666	0.55712923	0.90629275	-0.941604	-0.9770487

Cluster 3

Gene	A477	A490	A500	A575	Sum
C1QA	-1.2131706	0.70929385	0	-0.6853385	-1.1892152
C1QB	-1.4545016	0.92044889	0	-0.5245749	-1.0586276
C1QC	-0.7274363	0.54090872	0	-0.4797037	-0.6662314
CD8B	1.04708342	0.38218292	0	0.56822356	1.9974899
EIF5A	0.55938091	0.32274458	0	0.26006789	1.14219338
FTL	-1.9336857	0	0.56120984	-0.8718575	-2.2443333
GZMK	0.9268166	0.42716221	0	0.34942983	1.70340864
HLA-B	0.33190937	0.51518903	-0.5378983	-0.4223575	-0.1131574
HLA-DRB5	0.78828352	0.2540163	0	0.42631103	1.46861085
ITGB2	0.39410116	0	0.70829096	-0.4548319	0.64756025
LIMD2	0.72247461	0.30331254	0	0.38857932	1.41436646
MARCO	-0.790497	0.97097808	0	-0.750415	-0.5699339
MT-ATP6	0.27986832	-0.6683285	-1.0977349	-0.2538844	-1.7400795
MT-CO2	0.30045667	0	-0.539201	-0.346063	-0.5848074
MT-CO3	0.29573124	0	-0.4023068	-0.5071874	-0.613763
MT-CYB	0.47682915	-0.3073948	-0.4380092	-0.3139692	-0.582544
MT-ND4	0.33183797	0	-0.8472615	-0.4924933	-1.0079168
MT-ND5	0	-0.5559325	-0.7520771	-0.5590515	-1.8670611
PSAP	-0.9208013	0.46988511	0	-0.5441085	-0.9950247
RPL27A	-0.8211974	-0.3409284	-0.7221544	-0.3608842	-2.2451644
RPS26	0	0.34013921	0.42588558	-1.8830674	-1.1170426
S100A8	-1.3350353	1.03595572	1.63462225	-0.6124302	0.72311248
S100A9	-1.7742481	0.73908081	1.15073192	-0.7709904	-0.6554258
TMSB4X	0	-0.2902321	-0.9170042	-0.2969595	-1.5041958
TRGC2	0.40224775	0.29402121	0	0.36725718	1.06352614
VAMP2	0.74234878	0.3922278	0	0.43923184	1.57380842

Cluster 4

Gene	A477	A490	A500	A575	Sum
TUBA1A	0.93035577	0.96285887	0.92071227	0	2.81392691
TAGLN2	0.91976085	0.69098449	0.44068244	0	2.05142778
LILRB2	0.62519898	0.57380822	0.78008371	0	1.97909091
S100A6	0.66342736	0.83915708	0.74164779	-0.282838	1.96139424
UCP2	0.43577568	0.95163608	0.53742159	0	1.92483334
PPDPF	0.51782582	0.56687415	0.78605299	0	1.87075295
TIMP1	0	0.54245043	1.51615603	-0.3718143	1.68679219
KLF2	0.96980427	0	0.33497999	0.3307733	1.63555757
SERF2	0.28377944	0.63830132	0.69471997	0	1.61680073
EMP3	0.58748154	0.34807103	0.67634844	0	1.61190102
SPI1	0.48107567	0.67754987	0.43718264	0	1.59580818
FOS	2.25677692	0	-0.2946765	-0.4469138	1.51518659
GLUL	0	0.77264547	1.09878449	-0.3675504	1.50387954
HLA-DRB5	1.323126	0	-1.4813211	1.62682054	1.46862544
MYO1G	0.2678654	0.57958155	0.61974958	0	1.46719653
CYP1B1	0	0.99893598	1.01670029	-0.5561525	1.45948377
SLC39A8	0	0.8418358	1.08038183	-0.5182512	1.40396639
RHOG	0.46598007	0.36185796	0.52829845	0	1.35613648
CD55	0.63916249	0	1.00937964	-0.3060006	1.34254151
S100A4	0.36512555	0.64011889	0.33441071	0	1.33965514
UQCR11	0.36310269	0.49326247	0.46571312	0	1.32207829
ZFP36L1	0.70164982	0.268899	0.30583684	0	1.27638566
CALM3	0	0.75940126	0.68520259	-0.2735634	1.17104042
CTSB	0	0.72875024	0.88176589	-0.4468371	1.16367905
TYROBP	0	0.41984775	0.46312242	0.27818683	1.16115701
S100A8	0	1.33663373	1.50551999	-1.7460757	1.09607797
LGALS1	0	0.87989266	0.53617604	-0.3257016	1.09036714
LGALS9	0.46824455	0.49142486	0.3830148	-0.2808367	1.06184751
NAMPT	0.56691077	0	0.88603773	-0.4180879	1.0348606
FPR1	0	0.77195505	0.75771625	-0.5049112	1.02476011
LMNA	0.87026822	0	0.43283024	-0.287101	1.01599745
FTH1	0.36842514	0	0.95296989	-0.3452023	0.97619275
SMIM25	0	0.67062218	0.70622003	-0.4040213	0.97282089
STXBP2	0	0.72179665	0.64514564	-0.4146863	0.95225598
CD163	0	0.50503918	0.91941369	-0.504152	0.92030084
P4HB	0	0.65764433	0.48654156	-0.2828672	0.86131874
SH3BGRL3	0.36401488	0.56155523	0.29327777	-0.4209472	0.79790067
TUBB	0	0.76085302	0.3362902	-0.3027437	0.7943995

NEAT1	0.4429573	0	0.69626021	-0.364821	0.77439646
MAFB	0.50614938	0	0.70364912	-0.4622809	0.74751756
FLNA	0.536526	0.49369025	0	-0.3131033	0.71711295
ANXA2	0.50943427	0.62330266	0	-0.4231966	0.70954035
CALR	0	0.38063135	0.6570477	-0.3487126	0.68896646
EFHD2	0	0.53838119	0.49741605	-0.3533498	0.68244747
SDF2L1	0	0.38266594	0.60297206	-0.3362248	0.64941322
FKBP1A	0.51907224	0.4091183	0	-0.2792435	0.64894706
DDX21	0	0.40466645	0.52699455	-0.282782	0.64887899
MS4A7	0.53082039	0	0.40491458	-0.2953511	0.64038384
ITGB2	0.454242	0	0.54723711	-0.3655837	0.63589537
BLVRA	0	0.39634606	0.48191776	-0.2846651	0.59359877
BLVRB	0	0.57816579	0.32746645	-0.3321677	0.57346458
GAPDH	0	0.45884377	0.31269081	-0.2579532	0.51358139
VCAN	0	0.66519385	0.95255148	-1.1339543	0.48379103
AGFG1	0	0.26992	0.62206361	-0.4095157	0.48246793
VIM	0.37626449	0.318568	0	-0.2786895	0.41614298
LIPA	0.42545312	0.33075605	0	-0.42813	0.32807912
PDK4	1.28018702	0	-0.3457989	-0.6377446	0.29664346
POLR2L	0	0.47906503	0.30813929	-0.4935668	0.29363748
CTSL	0	0.39436251	0.39761522	-0.5150861	0.27689165
TYMP	0.53008588	0	0.45359924	-0.7629171	0.22076801
FCGR3A	0	0.29432523	0.35445572	-0.5585239	0.09025708
F13A1	-0.8248991	0	0.56983461	0.340658	0.08559356
RPS26	0.32046065	0.49884155	0.40667967	-1.152744	0.07323789
WARS	0.6977914	-0.3930495	-0.2572964	-0.2905519	-0.2431064
GBP4	0.39194568	-0.3504107	-0.3164482	0	-0.2749133
HLA-DOA	0	-0.3501894	-0.2705488	0.33656176	-0.2841764
PSME2	0.61853777	-0.4376003	-0.25631	-0.2962525	-0.3716251
HLA-C	0.56414125	-0.4052302	-0.2850216	-0.2696116	-0.3957221
RPL19	0	-0.2851343	-0.3875017	0.25058336	-0.4220526
MX1	0.32850796	-0.4133077	0	-0.3413313	-0.4261311
S100A9	0	0.75701478	0.39823831	-1.5888371	-0.433584
RPL18	0	-0.2623757	-0.4656293	0.2538854	-0.4741196
RPL30	0	-0.3416862	-0.4245173	0.28581411	-0.4803894
RACK1	0	-0.2715309	-0.5543987	0.29087849	-0.5350511
RPS3	0	-0.3268519	-0.6654676	0.39435236	-0.5979671
RPL10	0	-0.3266291	-0.6316775	0.33700674	-0.6212999
A2M	0	-0.540132	-0.6194579	0.4992365	-0.6603534
RPS23	0	-0.3838343	-0.6265157	0.33137729	-0.6789727
RPL5	-0.3621615	0	-0.7121026	0.39528811	-0.678976

LPAR6	0	-0.3521972	-0.6986878	0.35332546	-0.6975596
NOP53	0	-0.4136499	-0.5997467	0.2786783	-0.7347183
LTA4H	0.59311961	-0.5456278	-0.3646782	-0.4407484	-0.7579348
RPL6	-0.325133	-0.2529242	-0.5163419	0.3131592	-0.7812399
RPL11	-0.3044793	-0.2715154	-0.5317536	0.32565284	-0.7820955
GBP1	0.57991367	-0.5985633	-0.475429	-0.3509669	-0.8450455
RPS9	-0.2860319	-0.2714351	-0.3108164	0	-0.8682834
RPS14	-0.2592131	-0.2548097	-0.3878755	0	-0.9018983
RPL9	0	-0.3821218	-0.7839262	0.25548203	-0.9105659
RPS24	-0.3575199	-0.2904667	-0.5230464	0.25107897	-0.919954
ZFP36L2	0	-0.6895163	-0.7476236	0.42584004	-1.0112999
CLEC12A	0	-0.5030674	-0.2516512	-0.2860325	-1.0407511
RPL13	-0.3498303	-0.2931134	-0.4832815	0	-1.1262252
MT-CO3	0	-0.2875999	-0.5167991	-0.3226573	-1.1270563
RPL7A	-0.3667497	-0.4122831	-0.7008129	0.33297801	-1.1468678
EEF1A1	0	-0.5456297	-1.0473648	0.42865172	-1.1643428
ALOX5AP	-0.4922645	0.32299834	-1.026621	0	-1.1958872
RPL21	-0.3243971	-0.2759263	-0.5956767	0	-1.1960001
RPS7	-0.3795812	-0.358229	-0.5053811	0	-1.2431913
RPS3A	-0.2849615	-0.4341765	-0.8318526	0.28327373	-1.2677168
RPS11	-0.3424766	-0.4744528	-0.4872704	0	-1.3041998
TPT1	-0.3475728	-0.3913971	-0.5692212	0	-1.3081911
HLA-DMA	0	-0.8373954	-0.8156998	0.32652319	-1.3265719
RPL23	-0.3605431	-0.4578133	-0.5126067	0	-1.3309631
STAT1	0	-0.6570932	-0.4237978	-0.2853882	-1.3662792
RPL14	-0.347686	-0.3784869	-0.6962528	0	-1.4224257
MSR1	0	-0.397587	-0.6036252	-0.4445102	-1.4457225
RPS4X	-0.4126876	-0.5457232	-0.7915419	0.2884612	-1.4614915
CD74	0	-1.1656533	-0.8692165	0.55846693	-1.4764029
RPL7	-0.3232584	-0.3869333	-0.7702211	0	-1.4804128
RPL13A	-0.4034883	-0.4645587	-0.6295818	0	-1.4976287
HLA-DQB1	0	-1.0359287	-0.9020399	0.43957317	-1.4983955
RPL15	-0.2862652	-0.4672162	-0.7680454	0	-1.5215268
CST3	0	-1.0866551	-1.0523707	0.53642925	-1.6025966
RPS18	-0.3381476	-0.4711581	-0.8070391	0	-1.6163448
TMSB4X	0	-0.3927469	-1.0584721	-0.2595567	-1.7107757
RPS6	-0.3622702	-0.4792786	-0.8747076	0	-1.7162564
CFD	-1.3091518	-0.7433435	0.33414538	0	-1.7183499
RPL27A	-0.492805	-0.5315781	-0.7372393	0	-1.7616224
RPL3	-0.5437133	-0.543452	-0.8533016	0	-1.9404669
LYZ	-0.7980419	0	-0.6490073	-0.522322	-1.9693712

PLA2G16	-0.6903641	-0.4847897	-0.8218796	0	-1.9970334
HLA-DRA	0	-1.7997566	-1.3681889	0.54535321	-2.6225922
HLA-DQA1	0	-1.4538895	-1.6441495	0.25792875	-2.8401102
HLA-DPA1	0	-2.0897991	-1.5942884	0.71922168	-2.9648658
HLA-DPB1	0	-2.356919	-1.5461547	0.72292038	-3.1801533
HLA-DRB1	-0.8867089	-1.7117399	-1.4985539	0	-4.0970027

Cluster 5

Gene	A477	A490	A500	A575	Sum
CCL4	0.9900961	1.10073067	0	0.40200105	2.49282781
CD7	1.03096936	0.47334757	0	0.42846159	1.93277851
CMC1	-0.9428524	-0.7357872	0	-0.6425295	-2.3211691
CYBA	0.43706218	0.66878798	0	0.29425511	1.40010527
FTL	-1.7989651	0	1.6809705	-0.8886751	-1.0066698
GSTP1	-0.8458329	-0.8799453	0	-0.6903052	-2.4160835
HLA-B	0.38894741	0.5330962	0	-0.4499375	0.47210608
MARCO	-0.9422415	0.99091825	0	-0.7021864	-0.6535097
MT-ATP6	0.38036827	-0.7086677	-0.8471537	0	-1.1754532
RPL27A	-0.5180394	0	-0.9921964	-0.4451494	-1.9553852
RPL3	-0.850409	-0.437949	0	-0.3461859	-1.6345439
RPS18	-0.6058098	-0.3486569	-0.6777074	0	-1.6321741
RPS26	0.54617324	0.6653011	0	-1.4641963	-0.2527219
S100A4	0.4849099	0.87738097	1.05764578	0.39897426	2.8189109
S100A6	0.42710017	0.89051558	1.19904432	0.36186869	2.87852876
S100A8	-0.7649939	1.25931118	2.38532999	0	2.87964726
S100A9	-1.3097438	0.97495907	1.87155169	-0.6851437	0.8516233
TPT1	-0.5559159	-0.3683859	-0.6485746	0	-1.5728764

Cluster 6

Gene	A477	A490	A500	A575	Sum
ANXA2	0.38913475	0.39605613	0.35315277	0	1.13834365
CD99	0	0.31355581	0.39527745	0.26436806	0.97320132
CLEC12A	-0.750705	-0.6871496	0	-0.4880058	-1.9258605
CRIP1	0.50402952	0.43100396	0.59185881	0	1.52689229
ENHO	0.87787525	-0.5979886	-0.5007652	0.58157848	0.36069992
ENO1	0.33474352	0.46315622	0.27888947	0	1.07678921
ERAP2	-0.6190863	-0.7054224	-0.311468	0	-1.6359767
F13A1	-0.7133817	0.7173841	0.7832502	0.27458093	1.06183357
FCER1G	0.60033488	0.5971639	0	-0.2609627	0.93653611
FCN1	0.63517461	-1.0142666	-0.8236112	0	-1.2027032
GAPDH	0.44772123	0.57783444	0.36095528	0	1.38651095
GATM	-0.68644	-0.6461141	-0.6408753	0	-1.9734294
GBP1	0.36656469	-0.396002	-0.3256165	0	-0.3550537
GLIPR2	0.43867923	0.46070816	0.33729728	0	1.23668467
HLA-B	0	-0.4382645	-0.5558925	-0.667585	-1.6617421
HLA-C	0	-0.7841369	-0.5965598	-0.5167566	-1.8974533
HLA-DPB1	0	-1.8293277	-1.0243848	0.66933088	-2.1843816
HLA-DQB1	0	-0.8359748	-0.320452	0.56078914	-0.5956376
HLA-DRB5	1.48870438	0	-1.2821275	1.32017467	1.5267515
HSPB1	0	0.26227977	0.38749245	0.35354252	1.00331474
IFITM3	0	0.36425144	0.87182841	0.3022138	1.53829364
IGHM	0	-0.742512	-0.6912063	-0.6901681	-2.1238864
IL6ST	0	-0.3972977	-0.3561877	-0.3031963	-1.0566817
LGALS9	0.35704581	0.56860735	0.34495323	0	1.27060639
LTA4H	0	-0.6064493	-0.5213446	-0.6397068	-1.7675007
MEF2C	0	-0.2716951	-0.2641567	0.32797462	-0.2078771
MGLL	0	-0.3114864	-0.3151834	-0.3136149	-0.9402847
MSR1	0	-0.302145	-0.2971563	-0.2967072	-0.8960086
MT-ND4L	-0.8060454	-0.6016091	-0.3834454	0	-1.7910999
NR1D2	0	-0.2584892	-0.4500218	-0.4466012	-1.1551123
NUCB1	0.37014188	0.42853718	0.47351833	0	1.27219738
NUPR1	-0.8784462	-0.2708302	0	-0.520337	-1.6696134
P4HB	0.40311362	0.80242333	0.25009744	0	1.45563439
PLA2G16	-0.5009983	-0.2634516	-0.3926835	0	-1.1571335
PPDPF	0.43246787	0.45312225	0.70871152	0.40669128	2.00099291
RHOC	0.31636829	0.25615459	0.29311851	0	0.86564138
RPL27A	-0.3028644	-0.4810094	-0.5304375	0	-1.3143113
RPL3	-0.4491375	-0.2640844	-0.4983094	0	-1.2115313

RPS26	0	0.50865683	0.36462925	-0.6372494	0.23603665
RPS3A	-0.2807145	-0.2553643	-0.6300737	0	-1.1661525
RPS4X	-0.362588	-0.3348655	-0.4601533	0	-1.1576068
S100A6	0.84985297	0.35153642	0.80605256	0	2.00744195
S100A8	0	0.9427154	1.39986067	-0.7344293	1.60814673
S100A9	0	0.7227081	0.84535818	-0.5922471	0.97581913
SERF2	0.27621036	0.56697519	0.59436932	0	1.43755487
SH3BGRL	-0.3601481	-0.2791001	-0.4196038	0	-1.058852
SLC39A8	0	0.32798504	0.6676176	-0.3703635	0.62523917
SPI1	0.38099905	0.38299357	0.33377602	0	1.09776864
STAB1	0	1.0181138	0.66653924	0.2859863	1.97063934
TAGLN2	0.72477741	0.58257774	0.41179577	0	1.71915092
TMEM258	0.34524958	0.45201485	0.32584675	0	1.12311118
TPT1	-0.3469302	-0.25316	-0.3972503	0	-0.9973405
TYMP	0.45230451	-0.3008455	0.52199267	-0.6168006	0.05665118
UCP2	0.53150231	0.77918802	0.43625973	0	1.74695006
UQCR11	0.47009644	0.56184491	0.44549267	0	1.47743402
ZFP36L2	0	-0.4042407	-0.5701648	0.32574061	-0.6486649
ZYX	0.54186652	0.26303874	0	0.26561792	1.07052317

Cluster 7

Gene	A477	A490	A500	A575	Sum
C1QA	-0.8858723	0.51786746	0	-0.7324463	-1.1004512
C1QB	-1.171314	0.49274371	0	-0.6907032	-1.3692736
HLA-B	0.33977013	0.35413411	-0.7797071	-0.4757257	-0.5615286
MT-ATP6	0	-0.5796748	-1.2656393	-0.2571249	-2.102439
MT-CO1	0.3419961	-0.607879	-0.6213787	-0.3117342	-1.1989957
MT-ND2	0	-0.2800506	-1.4645894	-0.3870397	-2.1316796
MT-ND4	0.35009759	0	-0.7768889	-0.4702342	-0.8970254
RPS19	-0.3839035	-0.3092497	0	-0.3105984	-1.0037516
S100A8	-1.0865819	0.73425384	2.0356572	-0.6790132	1.00431587
S100A9	-1.4685725	0.3663484	1.19687403	-0.9266971	-0.8320472
TMSB4X	0	-0.5055346	-1.1644306	-0.2956732	-1.9656384
VAMP2	0.5563992	0.29896649	0	0.36985096	1.22521665

Cluster 8

Gene	A477	A490	A500	A575	Sum
ALDH2	0	-0.9851804	-0.6769758	-0.5289349	-2.1910911
C1QB	0	-0.5966877	-0.5618853	-0.9725925	-2.1311655
C1QC	0	-0.8391519	-0.6738183	-0.8596285	-2.3725988
CALM2	0	-0.6015425	-0.5996375	-0.2638717	-1.4650518
CEBPB	0	-0.9124803	-0.6739658	-0.3128653	-1.8993115
CFD	-1.5252036	-2.3300229	-0.9195766	-0.9398514	-5.7146545
CST3	0	-1.5944491	-1.0280451	-0.7546697	-3.3771638
DDX5	-0.3485399	-0.600258	-0.2600773	0	-1.2088752
EPB41L2	-0.6832752	-0.547555	-0.5177892	-0.589758	-2.3383774
EPS8	-0.7463869	-0.452303	-0.4659421	0	-1.6646319
FCGR3A	0	-0.8134379	-0.8316869	-0.7416612	-2.3867859
FOLR2	0	-0.5488183	-0.2969679	-0.4499591	-1.2957453
GATM	-0.4182018	0	-0.5053549	-0.2531171	-1.1766737
HLA-B	0	-0.4949122	-0.787971	-0.5570878	-1.839971
HLA-DRB1	0	-1.5881269	-0.4642771	-0.9471233	-2.9995273
IFI27	0	-0.9647389	-0.4935688	-1.1418219	-2.6001296
LTA4H	0	-0.7602816	-0.7207922	-0.8426776	-2.3237514
LYVE1	0	-0.5677248	-0.4783857	-0.4907653	-1.5368759
LYZ	-0.6212568	-0.7783519	0	-0.3422575	-1.7418663
MGLL	0	-0.7944947	-0.7552524	-0.5345141	-2.0842611
MT-ATP6	-1.3929912	-0.6369022	-1.333368	0.77181508	-2.5914464
MT-CO2	-1.7868425	-0.5356565	-1.0767346	0	-3.3992336
MT-CO3	-1.5195495	-0.6929545	-1.0931841	0	-3.3056882
MT-CYB	-2.0686548	-0.6315808	-0.892023	0	-3.5922586
MT-ND1	-1.931918	-0.5341609	-0.6569345	0	-3.1230134
MT-ND2	-2.8983042	-0.9865238	-1.3477437	0	-5.2325718
MT-ND3	-1.9696558	0	-0.7536173	0.67615069	-2.0471225
MT-ND5	-1.9010207	-0.9396361	-1.0929299	0	-3.9335867
N4BP2L2	-0.3262886	-0.6037912	-0.6461274	0.41009196	-1.1661152
NUPR1	-0.8550681	-0.781216	-0.5743631	-0.7627587	-2.9734059
PTPRC	-0.5156095	0	-0.4714285	0.68410894	-0.3029291
RPL28	0	0.61145223	0.54851859	0.49288945	1.65286027
RPL36	0	0.39749858	0.566298	0.35083578	1.31463235
RPL41	0	0.55672315	0.55050642	0.49761763	1.60484719
RPS15	0	0.44739229	0.60750037	0.3036463	1.35853896
RPS26	0	0.87052982	0.94994656	-0.78332	1.03715643
RPS28	0	0.28987015	0.49027779	0.29697166	1.0771196
RPS8	0	0.58891948	0.61879141	0.33390355	1.54161444

SAT1	0	-0.2861895	-0.4115614	-0.3264567	-1.0242077
SELENOP	0.25939462	-0.3463492	-1.1275059	0	-1.2144605
SERF2	0.47106122	0.41779007	0.61945026	0	1.50830155
SNHG8	-0.3957939	0	-0.512562	0.51943209	-0.3889238
TXNIP	0	-0.4223686	-0.7261874	0.49057312	-0.657983
ZFP36L2	0	-0.4304861	-0.8622655	0.49662517	-0.7961264

Cluster 9

Gene	A477	A490	A500	A575	Sum
HLA-A	0.65838835	0	0.37425285	0.293321	1.3259622
HLA-DRB1	-1.1784467	0	0.4066057	-0.6220679	-1.3939089
RPL27A	-0.4716014	-0.5462826	-0.611536	0	-1.62942
RPS26	0.38600589	0	0.35760285	-1.6652076	-0.9215988
S100A8	-1.4486871	1.7611659	1.52412539	-1.1982678	0.63833639
S100A9	-1.372472	1.69581418	0.93164225	-1.7233429	-0.4683584

Cluster 10

Gene	A477	A490	A500	A575	Sum
ALDH2	0	-1.0379822	-1.0291606	-0.389831	-2.4569737
CFD	-1.717751	-2.2295644	-1.7887138	-0.8373915	-6.5734207
CST3	0	-1.4182501	-1.9716396	-0.4498147	-3.8397044
GAPDH	0.84650074	1.07479752	0.55846385	0	2.47976211
HLA-DRB1	0	-1.2638501	-1.7351486	-0.4134738	-3.4124725
LGALS1	0.95438678	1.44686045	1.24543363	0	3.64668086
PPDPF	0.6995447	0	0.91941685	0.47479961	2.09376116
RPS26	0	0.61663445	0.4823285	-0.8503819	0.248581
UCP2	0.73686894	0.84547808	0.49783664	0	2.08018366

Cluster 11

Gene	A477	A490	A500	A575	Sum
HLA-B	0	0.48134876	-0.6178308	-0.6718282	-0.8083103
S100A8	0	0.67549981	1.26926806	-0.9263083	1.0184596

Cluster 12

Gene	A477	A490	A500	A575	Sum
S100A9	-1.1525484	0	0.67458983	-0.6435213	-1.1214799

Cluster 13

Gene	A477	A490	A500	A575	Sum
CD27	0	0.61904686	0.79939318	0.63906935	2.05750939
TMSB4X	0	-0.6351634	-1.2976827	-0.353158	-2.2860041

Cluster 14

Gene	A477	A490	A500	A575	Sum
CD74	0	-0.8599729	-0.6329624	0.45021599	-1.0427194
RPS26	0	0.64919806	0.45726633	-0.7209211	0.38554325
TAGLN2	1.17510947	0.87672417	0.56508874	0	2.61692238

Cluster 15

	A477	A490	A500	A575	Sum

Cluster 16

	A477	A490	A500	A575	Sum

Cluster 17

	A477	A490	A500	A575	Sum

Cluster 18

	A477	A490	A500	A575	Sum

	WT(Cst3 $^{fl/fl}$) n=7		Cst3 $^{\Delta Lyz2}$ n= 5		
Parameter	Mean	SD	Mean	SD	p value
HR (bpm)	400.40	27.44	391.00	54.01	0.698
SW (mmHg*µL)	1254.41	274.65	1098.00	182.08	0.296
CO (µL/min)	7610.00	1284.52	6917.00	762.13	0.309
SV (µL)	19.03	3.13	17.82	1.89	0.462
EF (%)	33.65	5.80	37.63	9.29	0.380
Ea (mmHg/µL)	4.64	1.26	4.75	1.02	0.877
PowMax (mmHg*µL/s)	24245.71	7840.00	34040.00	19090.20	0.225
dP/dt max (mmHg/s)	5775.00	621.13	5378.00	825.86	0.362
dP/dt min (mmHg/s)	-4345.71	663.10	-4598.00	716.03	0.543
Tau (ms)	9.44	2.53	9.81	1.76	0.783
PRSW	33.39	8.98	33.24	11.63	0.967
EDPVR	0.32	0.09	0.24	0.10	0.260
ESPVR	2.71	1.73	2.51	1.06	0.825

Supplemental Table 4. Left ventricle Pressure-Volume Loop assessment at baseline in WT(Cst3$^{fl/fl}$) or Cst3$^{\Delta Lyz2}$ mice. HR-heart rate, SW-stroke work, CO-cardiac output, SV- stroke volume, EF- ejection fraction, Ea-Arterial elastance, PowMax- maximal power, dP-delta pressure, dV-delta volume, PRSW- preload recruitable stroke work, EDPVR- end diastolic pressure volume relationship, ESPVR-end systolic pressure volume relationship.

	WT (Cst3 $^{fl/fl}$) (n=14)		Cst3 $^{\triangle Lyz2}$ (n=15)		
Parameter	Mean	SD	Mean	SD	p value
HR (bpm)	445.31	45.25	503.12	41.75	**0.001**
SW (mmHg*µL)	1059.23	363.07	800.17	316.12	**0.049**
CO (µL/min)	7507.14	1814.53	7227.67	2649.13	0.745
SV (µL)	17.07	4.76	14.41	5.32	0.168
EF (%)	29.25	10.37	24.86	12.93	0.324
Ea (mmHg/µL)	5.37	1.45	6.73	2.33	0.073
PowMax (mmHg*µL/s)	35227.14	36756.23	36527.53	19857.69	0.905
dP/dt max (mmHg/s)	5829.79	763.15	5859.00	817.81	0.921
dP/dt min (mmHg/s)	-4592.79	882.75	-4359.60	651.95	0.423
Tau (ms)	9.33	1.18	10.25	1.91	0.137
PRSW	41.72	12.27	27.30	14.83	**0.011**
EDPVR	0.34	0.13	0.51	0.23	**0.032**
ESPVR	4.06	1.05	3.15	1.14	**0.036**

Supplemental Table 5. Left ventricle PV Loop assessment 4 weeks post-MI in WT (Cst3$^{fl/fl}$) and Cst3$^{\triangle Lyz2}$ mice. MI- myocardial infarction, HR-heart rate, SW-stroke work, CO-cardiac output, SV- stroke volume, EF- ejection fraction, Ea-Arterial elastance, PowMax- maximal power, dP-delta pressure, dV-delta volume, PRSW- preload recruitable stroke work, EDPVR- end diastolic pressure volume relationship, ESPVR- end systolic pressure volume relationship.
